# Estimates of the global burden of HIV-attributable and all-cause AIDS-defining cancers: a population-based modeling study

**DOI:** 10.3389/fpubh.2026.1933709

**Published:** 2026-09-10

**Authors:** Xiaojun Meng, Bolin Yang, Jibiao Chen, Hanlu Yin, Zhuping Xu, Jing Gu, Wenjuan Ma, Yuan Shen

**Affiliations:** The Affiliated Wuxi Center for Disease Control and Prevention of Nanjing Medical University, Wuxi, Jiangsu, China

**Keywords:** AIDS-defining cancers, global burden, people living with HIV, population attributable fraction, risk ratio

## Abstract

**Introduction:**

People living with human immunodeficiency virus (HIV; PLHIV) have elevated risks of AIDS-defining cancers (ADCs), including cervical cancer, non-Hodgkin lymphoma (NHL), and Kaposi sarcoma (KS). Previous studies have quantified the individual global burden of these cancers, we aimed to estimate the pooled global burden of HIV-attributable and all-cause ADCs in 2022 and identify the key drivers across different regions and countries.

**Methods:**

We conducted a systematic review and meta-analysis to update risk ratios (RRs) for cervical cancer, NHL, and KS. RRs were used to calculate population-attributable fractions (PAFs) and age-standardized incidence rates (ASIRs) of HIV-attributable ADCs using country-specific HIV prevalence and 2022 cancer incidence data. Associations with the Human Development Index (HDI) were assessed.

**Results:**

In 2022, an estimated 101,835 (95% CI 76102–131,298) ADC cases were attributable to HIV, accounting for 8.1% (95% CI 6.2–10.4) of all-cause ADC cases. NHL comprised 62.9% of HIV-attributable ADCs, while cervical cancer accounted for 53.0% of all cases. The highest HIV-attributable ASIRs were in Eswatini (34.9%), Zambia (32.0%), and Mozambique (25.9%). In East and Southern Africa, 27.8% of ADCs were linked to HIV. Among 35 countries with high cancer burden and HIV attribution, 68.6% were in Africa. Lower HDI correlated with higher PAF (*r* = −0.27), all-cause ASIR (*r* = −0.25), and HIV-attributable ASIR (*r* = −0.28).

**Conclusion:**

HIV significantly contributes to the global ADC burden. Addressing immunosuppression, viral co-infections, and healthcare access through ART expansion, oncogenic virus monitoring, and cancer prevention is essential to mitigate the dual burden of HIV and ADCs globally.

## Introduction

According to the latest global report, 39.9 million people globally were living with human immunodeficiency virus (HIV) and 630,000 died from AIDS-related illnesses in 2023 (1). People living with HIV (PLHIV) face a high risk of opportunistic infections and cancers, due to immunosuppression, which impairs the elimination of oncogenic viral infections (2, 3). Cervical cancer, non-Hodgkin lymphoma (NHL), and Kaposi sarcoma (KS) are the most common AIDS-defining cancers (ADCs) among PLHIV (4). While antiretroviral therapy (ART) has significantly reduced ADC incidence and mortality in high-income countries (5, 6), risks remain high in low- and middle-income countries (LMICs) (7, 8). Compared to the general population, PLHIV have markedly higher rates of cervical cancer, NHL, and KS (9), driven by chronic immunosuppression, persistent co-infection with oncogenic viruses, and sustained HIV viremia.

Cervical cancer is usually caused by persistent infection with high-risk human papillomavirus (HPV) genotypes, particularly HPV 16 and 18. It is the second leading cause of cancer-related death among women globally (10), with 90% of deaths occurring in LMICs, highlighting significant healthcare disparities and the prevalence of related risk factors (11). Although HIV does not directly cause cervical cancer, co-infection with HPV significantly increases the risk of cervical cancer through immunosuppression, impaired HPV clearance, and accelerated carcinogenesis (12, 13). A meta-analysis and modeling study reported that women living with HIV have a six fold higher risk of cervical cancer compared to the general population (14). Moreover, 4.9% of global new cases in 2018 were attributed to HIV infection, with southern and eastern Africa being the most affected regions (14). Integrating HIV and cervical cancer control strategies is crucial in high-burden regions.

NHL, the most common lymphoma subtype, accounts for approximately 90% of all lymphoma cases (15). According to the GLOBOCAN database, NHL ranked 10th and 12th in cancer incidence for males and females, respectively, in 2022 (16). NHL is linked to viral infections, such as Epstein–Barr virus (EBV) and human T-cell leukemia/lymphoma virus type I (HTLV-I). HIV-induced immunosuppression facilitates the replication of these viruses, thereby increasing the risk of developing NHL (17). Despite significant ART-related reduction in incidence, HIV-associated NHL remains a major cause of morbidity and mortality in PLHIV (18). A population-attributable modeling study estimated that PLHIV have a 24-fold higher NHL risk than in people without HIV, with approximately 7.0% of newly diagnosed NHL cases attributable to HIV infection in 2019 worldwide, with the highest in southern and eastern Africa (19). Due to disparities in HIV prevalence and ART coverage, geographic variations in NHL-associated HIV burden complicate global resource allocation and utilization.

KS is closely related to Kaposi sarcoma-associated herpesvirus (KSHV) and remains one of the most prevalent cancers in PLHIV (5). While KSHV infection is necessary for KS development, HIV-induced immunosuppression significantly increases the risk for KSHV co-infection and subsequent development of cancer (20). KS is rare in the general population (1.5 per 100,000 person-years) (21), but markedly more common in PLHIV with incidence rates per 100,000 person-years reaching 52 in Asia-Pacific, 180 in Europe, 237 in North America, 244 in Latin America, and 280 in South Africa (22). Nearly three-quarters of global KS cases occur in sub-Saharan Africa, home to approximately 25.5 million PLHIV, accounting for about 70% of the global total (1). In 2020, 71.4% of KS cases were attributed to HIV, with more than two-thirds of cases living in southern and eastern Africa (23).

As mentioned above, previous studies have quantified the individual global burden of these cancers (14, 19, 23), thereby establishing critical baseline estimates and underscoring the role of HIV-driven immunosuppression in accelerating oncogenesis across different anatomical sites and viral co-infection pathways. However, because these analyses examined each cancer in isolation, the pooled burden of all three ADCs combined remains unquantified. Their overlapping geographic distributions and shared mechanistic drivers—chronic immunosuppression, oncogenic viral co-infection, and limited ART access—have not been evaluated as an integrated cluster, preventing direct comparison of each ADC’s relative contribution to the overall HIV-attributable cancer burden. Analyzing these three AIDS-defining malignancies as a combined entity offers distinct epidemiological value. Given the overlapping risk factors and determinants, analyzing ADCs—cervical cancer, NHL, and KS—as a cluster would quantify the total ADC burden attributable to HIV, identify regional patterns transcending individual cancer sites, and inform integrated interventions targeting common upstream risk factors rather than single diseases. To address this gap, we conducted an updated literature review to revise risk ratios (RRs) for each ADC to a unified time point as of the end of 2024. Using 2022 global cancer data, we then estimated the pooled number of HIV-attributable ADCs and identified key drivers across UNAIDS regions and countries.

## Methods

### Literature review and study selection

To standardize burden estimates for the three ADCs (cervical cancer, NHL, and KS), we conducted updated literature reviews and meta-analyses to revise RRs for each cancer (14, 19, 23). Searches followed prior strategies and included six databases (Embase, PubMed, Web of Science, Cochrane Library, Global Index Medicus, and Global Health [CABI.org]), covering studies published through the end of 2024, with no language restrictions. For cervical cancer (International Classification of Diseases version 10th code C53) and NHL (C82-C86, C96), studies reporting the odds ratio (OR), RR, hazard ratio (HR), incidence rate ratio (IRR), and standardized incidence ratio (SIR) for the risk of specific cancers among PLHIV compared with HIV-negative people or the general population were retrieved with their corresponding 95% confidence intervals (CI). For KS, only studies reporting HIV prevalence among patients with KS were extracted.

Two independent reviewers screened and extracted data for each cancer: XJM and JBC (cervical cancer), HLY and BLY (NHL), and WJM and JG (KS). Study quality was assessed using the Agency for Healthcare Research and Quality (AHRQ) 11-item checklist (19), and categorized into high-, middle-, and low-risk levels corresponding to low, middle, and high quality, respectively. The corresponding author resolved disagreements.

### Data sources on HIV prevalence, related cancer cases, and incidence

HIV prevalence and ART data for adults (aged ≥15 years) in 2022 were collected from the UNAIDS website (1). Of the 205 UN member states, 172 with available data were included and categorized into eight UNAIDS regions; 31 countries lacking data were excluded. Age-specific incidence of newly diagnosed cancer cases and incidence rates for AIDS-defining cancers were retrieved from GLOBOCAN 2022, provided by the International Agency for Research on Cancer (IARC) (24). Combining these sources enabled statistical analysis of the global ADC burden.

### Statistical analysis

Details of the statistical methods used are shown in Supplementary Appendix 1. ORs, RRs, HRs, and SIRs were treated equivalently to estimate updated pooled RRs and 95% CI for cervical cancer and NHL through random-effects meta-analysis. These pooled RRs and country-specific HIV prevalence data were used to calculate the population-attributable fraction (PAF) using Levin’s formula. The cancer-specific RRs were modeled as log-normal distributions and HIV prevalence as beta distributions, each with 10,000 samples generated using R software (version 4.3.3). Subsequently, we calculated the country-specific PAF using Levin’s formula (25):

$\overset{Population\;attributable\;fraction}{\left(PAF\right)}$ = $\frac{\text{pHIV}\times (\text{pooled}\;\mathrm{RR}-1)}{1+\text{pHIV}\times (\text{pooled}\;\mathrm{RR}-1)}$

Where *pHIV* represents country-specific HIV prevalence. The total number of cervical cancer cases or NHL cases attributable to HIV in 2022 in a country was computed by multiplying the total number of new cases by the corresponding PAF. Because of the extremely high RR for KS among PLHIV, even in the ART era, HIV prevalence among patients with KS was assumed to be equivalent to PAF (23). Therefore, the total KS cases attributable to HIV for the year 2022 in a country were calculated by multiplying the total new KS cases by the pooled HIV prevalence among patients with KS.

The total incidence of ADCs attributable to HIV was calculated by summing HIV-attributable cases of each cancer. The combined PAF and the overall age-standardized incidence rate (ASIR) of ADCs attributable to HIV were estimated as follows:

PAF_AIDS-defining cancers_= $\frac{\text{Incidence number of AIDS}-\text{defining cancers attributable to}\;\mathrm{HIV}}{\mathrm{All}-\text{cause incidence number of AIDS}-\text{defining cancers}}$

ASIR _attributable to HIV_ = All-cause ASIR _AIDS-defining cancers_ × PAF_AIDS-defining cancers_

As no consensus standard currently exists, countries with all-cause ASIR above the global average were classified as high- and those below as low-cancer-burden countries. Similarly, countries with HIV-attributable PAF exceeding the global average were categorized as high HIV-attributable, and others as low HIV-attributable countries. Based on these criteria, countries were stratified into four distinct groups: (1) high cancer burden with high HIV attribution, (2) high cancer burden with low HIV attribution, (3) low cancer burden with high HIV attribution, and (4) low cancer burden with low HIV attribution. The associations between PAF, all-cause ASIR, and HIV-attributable ASIR in ADCs in PLHIV, as well as the Human Development Index (HDI), were evaluated using Spearman’s correlation tests.

The protocol of this systematic review was registered on the PROSPERO (CRD420250648591).

## Results

### RR and PAF for each ADC

For cervical cancer, 37 studies from 21 countries and six UNAIDS regions yielded a pooled RR of 4.85 (95% CI 3.69–6.37) for women living with HIV, with the highest RR in Asia and the Pacific at 9.83 (95% CI 4.24–22.80; Supplementary Tables S1, S4; Supplementary Figures S1, S2, S5). Globally, 2.6% (95% CI 1.8–3.6) of cervical cancer cases—ranging from 0.2% (95% CI 0.1–0.3) in the Middle East and North Africa to 12.8% (95% CI 8.9–17.2) in East and Southern Africa—were attributable to HIV (Table 1; Supplementary Figure S8), corresponding to an estimated 17,135 (95% CI 11637–24,029) patients in 2022 (Table 1). For NHL, the pooled RR of 21.14 (95% CI 15.86–28.19) among PLHIV was derived from 44 studies across 21 countries and 5 UNAIDS regions, with the highest RR of 27.67 (95% CI 19.27–39.74; Supplementary Tables S2, S4; Supplementary Figures S1, S3, S6) in Western and Central Europe and North America. In 2022, 64,068 NHL cases (95% CI 46, 886–84,877; 11.6% of global cases, 95% CI 8.6–15.2) were attributable to HIV (Table 1), ranging from 1.2% (95% CI 0.7–1.8) in the Middle East and North Africa to 21.1% (95% CI 15.9–27.3) in West and Central Africa (Table 1; Supplementary Figure S8). For KS, according to 92 studies from 41 countries and 6 UNAIDS regions, an estimated 57.8% (95% CI 49.6–65.7) of all cases were attributable to HIV—ranging from 13.7% (95% CI 0.7–35.6) in the Middle East and North Africa to 86.4% (95% CI 78.8–92.5) in East and Southern Africa—among PLHIV (Table 1; Supplementary Table S3; Supplementary Figures S1, S4, S7, S8), amounting to 20,632 (95% CI 9274–32,572) cases globally in 2022 (Table 1). Country-level PAF estimates for cervical cancer, NHL, and KS are shown in Supplementary Table S6.

**Table 1:** Estimated population attributable fraction of HIV for AIDS-defining cancers in 2022, by UNAIDS regions.

UNAIDS Region	Cervical cancer			Non-Hodgkin lymphoma		
	Number of new cases, 2022 (95% CI)	Population attributable fraction for HIV (95% CI)	Number of cases attributable to HIV (95% CI)	Number of new cases, 2022 (95% CI)	Population attributable fraction for HIV (95% CI)	Number of cases attributable to HIV (95% CI)
Global	662,042(633,772–690,191)	2.6%(1.8–3.6)	17,135(11,637–24,029)	553,156(525,513–581,070)	11.6%(8.6–15.2)	64,068(46,886–84,877)
Asia and the Pacific	386,992(357,704–416,433)	1.3%(0.4–3)	5,169(1,691–11,589)	220,546(195,985–244,733)	4.7%(3–6.9)	10,349(6,346–15,792)
Caribbean	3,891(3,207–4,566)	3.7%(2.5–5.3)	145(90–221)	2,468(100–4,806)	17.3%(12.8–22.6)	438(16–966)
Eastern Europe and Central Asia	31,933(30,529–33,362)	2.1%(1.4–2.9)	663(448–931)	18,770(16,488–21,037)	14.7%(11–18.9)	2,762(1,984–3,709)
East and Southern Africa	70,498(69,278–71,734)	12.8%(8.9–17.2)	8,997(6,229–12,154)	19,117(16,827–21,422)	18.8%(6.9–34.6)	3,590(1,298–6,689)
Latin America	58,906(55,575–62,253)	1.0%(0.7–1.4)	589(383–856)	39,990(32,161–47,817)	8.7%(6.4–11.5)	3,507(2,164–5,244)
Middle East and North Africa	11,844(11,330–12,360)	0.2%(0.1–0.3)	23(13–36)	28,071(26,305–29,813)	1.2%(0.7–1.8)	338(205–523)
West and Central Africa	46,197(41,221–51,153)	3.9%(1.4–7.6)	1,790(606–3,603)	14,552(1,052–29,966)	21.1%(15.9–27.3)	3,212(178–7,515)
Western and Central Europe and North America	51,619(50,273–52,960)	0.5%(0.3–0.7)	251(160–369)	208,873(206,001–211,700)	6.9%(4.6–9.8)	14,378(9,590–20,450)
	**Kaposi sarcoma**			**AIDS-defining cancers**		
Global	35,793(16,385–55,220)	57.8%(49.6–65.7)	20,632(9,274–32,572)	1,250,993(1,203,663–1,298,845)	101,835(76,102–131,298)	8.1%(6.2–10.4)
Asia and the Pacific	1,304(180–2,917)	26.9%(13.3–43.0)	350(42–895)	608,843(555,408–662,144)	15,868(8,449–27,516)	2.6%(1.4–4.4)
Caribbean	193(15–516)	57.8%(49.6–65.7)	112(8–300)	6,552(3,504–9,529)	696(207–1,291)	10.2%(5.5–14.6)
Eastern Europe and Central Asia	728(310–1,197)	57.8%(49.6–65.7)	420(177–701)	51,431(47,901–54,994)	3,845(2,822–5,064)	7.5%(5.7–9.6)
East and Southern Africa	21,649(7,214–36,441)	86.4%(78.8–92.5)	18,695(6,200–31,711)	111,264(95,884–127,010)	31,281(16,911–46,032)	27.8%(17.4–37.1)
Latin America	2,954(469–6,695)	62.2%(47.7–75.8)	1,840(292–4,269)	101,851(90,375–113,529)	5,937(3,628–8,936)	5.8%(3.9–8.2)
Middle East and North Africa	877(246–1,611)	13.7%(0.7–35.6)	120(4–442)	40,792(38,480–43,117)	481(267–840)	1.2%(0.7–2.0)
West and Central Africa	4,637(1,616–10,640)	72.0%(57.9–84.3)	3,336(1,137–7,703)	65,386(49,783–82,632)	8,338(3,231–15,111)	12.4%(6.3–19.2)
Western and Central Europe and North America	4,073(3,488–4,669)	43.2%(31.3–55.6)	1,760(1,224–2,337)	264,564(260,055–269,051)	16,389(11,454–22,570)	6.2%(4.3–8.5)

### Pooled PAF for ADCs and disparities in cancer-specific composition

**Figure 1:**
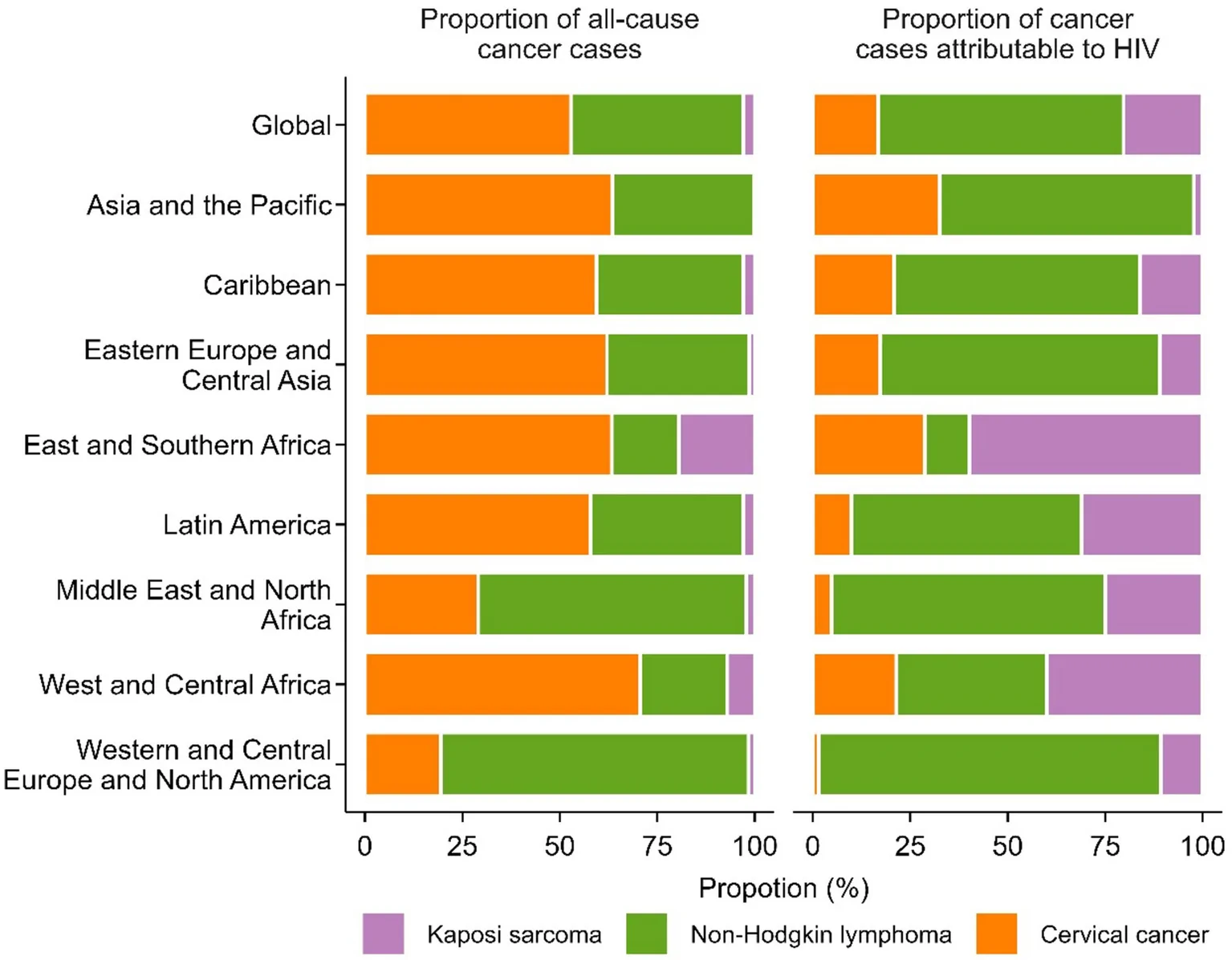
The distribution of incident cases of HIV-attributable and all-cause AIDS-defining cancers by UNAIDS regions and specific cancers. HIV, Human Immunodeficiency Virus. UNAIDS, Joint United Nations Program on HIV/AIDS.

In 2022, an estimated 1,250,993 new ADC cases were diagnosed worldwide—662,042 cervical cancer, 553,156 NHL, and 35,793 KS—accounting for 6.3% of all cancer cases. Additional analyses to estimate cancer burden in excluded countries having no HIV prevalence data were shown in the Supplementary Appendix 2. Of these, 8.1% (95% CI 6.2–10.4) or approximately 101,835 cases (95% CI 76102–131,298) were attributable to HIV (Table 1). Regional distributions ranged from < 5.0% in the Middle East and North Africa (1.2, 95% CI 0.7–2.0), and Asia and the Pacific (2.6, 95% CI 1.4–4.4), to the highest in East and Southern Africa (27.8, 95% CI 17.4–37.1). There were disparities in cancer-specific composition between all-cause and HIV-attributable cancer cases. Globally, NHL was the predominant contributor to HIV-attributable ADCs, whereas cervical cancer constituted the largest proportion of all-cause ADC cases (Figure 1). Across the UNAIDS regions, cervical cancer dominated all-cause ADCs in six regions, except in the Middle East and North Africa, and in Western and Central Europe and North America, where NHL was predominant. In contrast, KS was the leading HIV-attributable ADC in East and Southern Africa and West and Central Africa.

**Figure 2:**
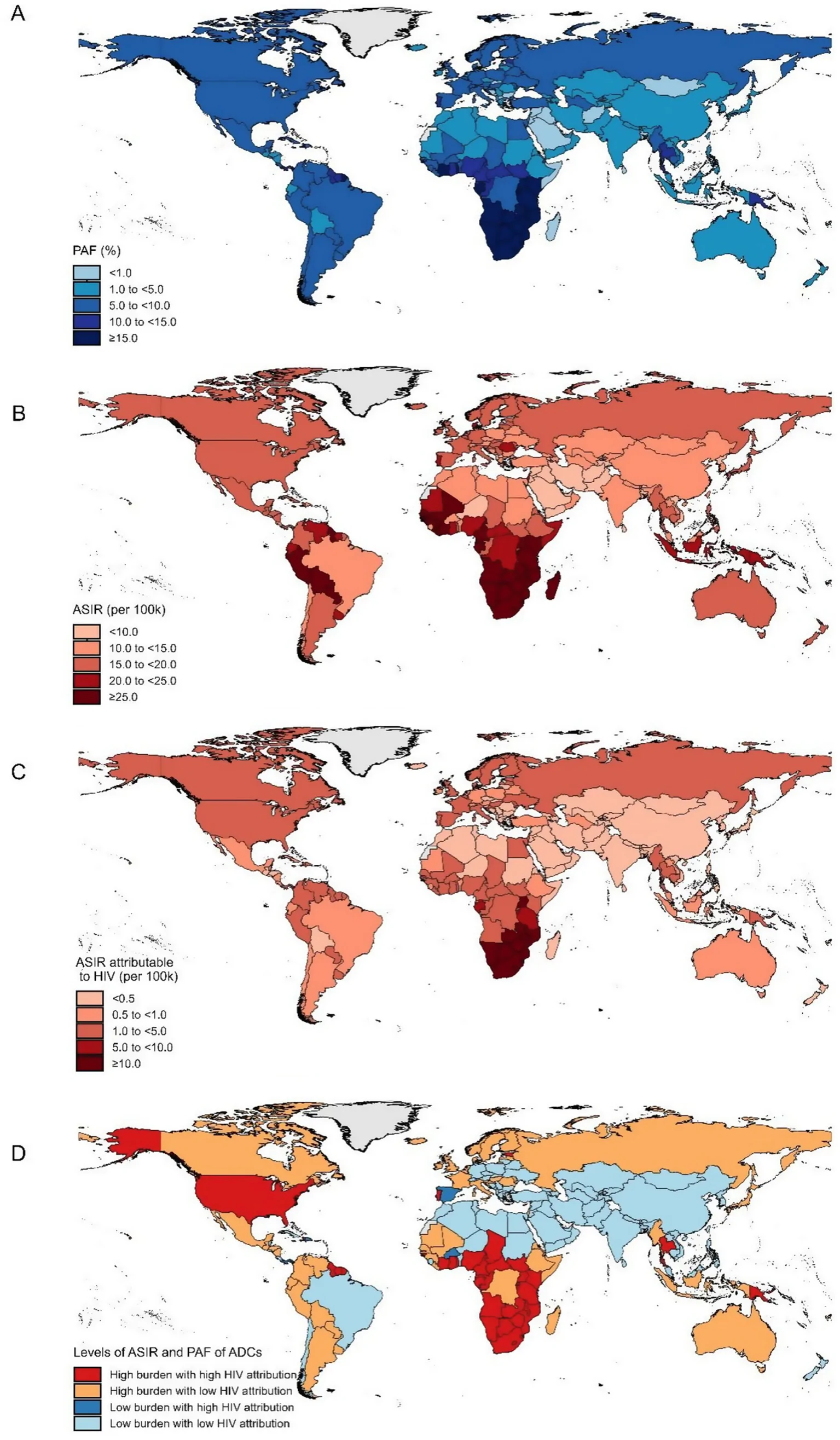
Country-specific PAF, HIV-attributable and all-cause ASIR, and classification of ADC burden. **(A)** Population attributable fraction (PAF) of HIV for AIDS-defining cancers (ADCs). **(B)** Age-standardized incidence rate (ASIR) of ADCs. **(C)** ASIR attributable to HIV infection. **(D)** Classification of ADC burden according to the levels of ASIR and PAF. HIV, human immunodeficiency virus.

**Figure 3:**
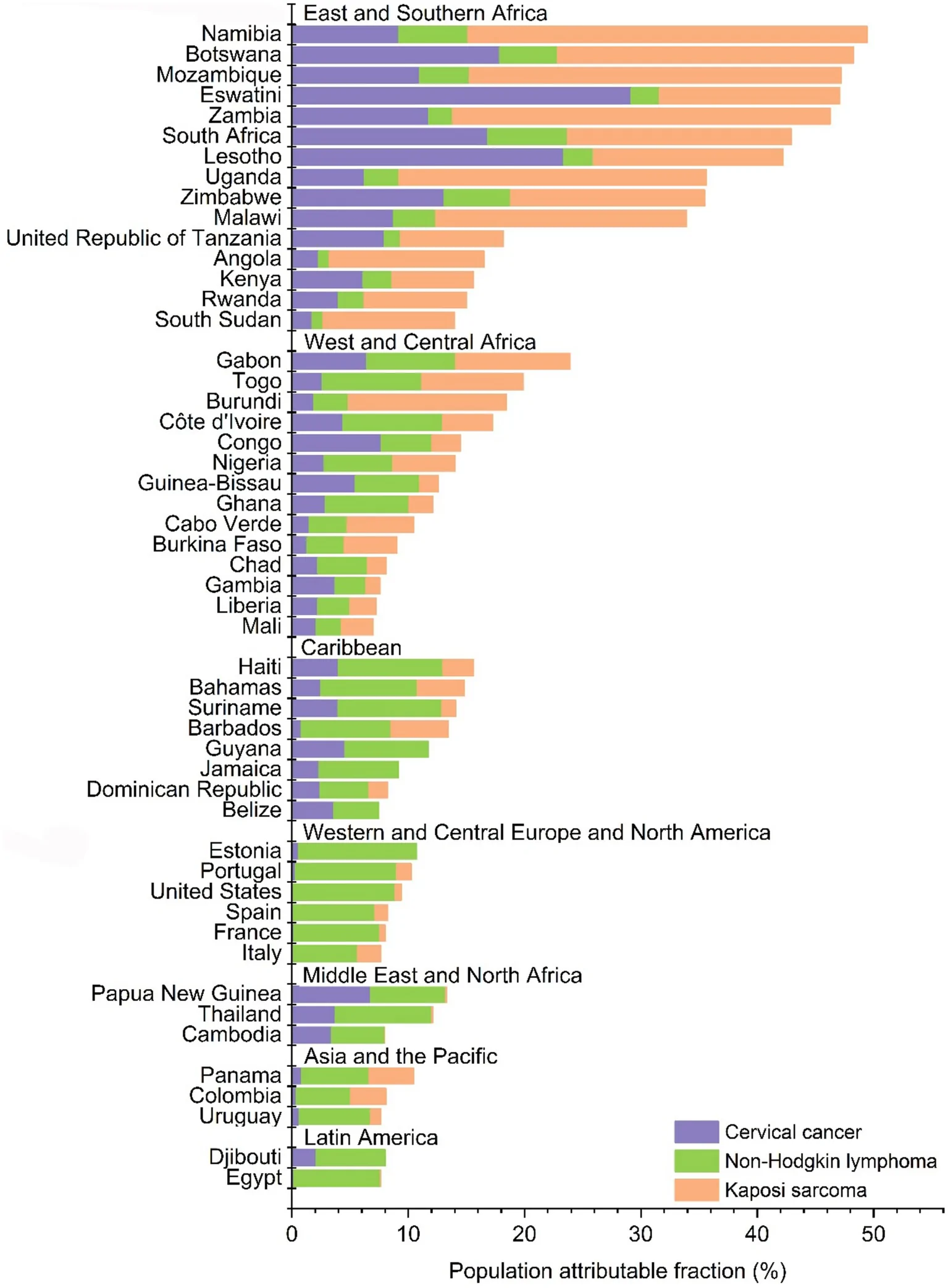
The contribution of each specific cancer to the population attributable fraction of HIV for AIDS-defining cancers in the 50 highest-ranked countries by UNAIDS regions. HIV, human immunodeficiency virus. UNAIDS, Joint United Nations Program on HIV/AIDS.

Among all countries analyzed, 41 had a PAF of ADCs attributable to HIV exceeding the global average of 8.1%, while 70 had a PAF below 5.0% (Figure 2A). The highest PAFs were observed in Namibia (49.5, 95% CI 37.5–60.0), Botswana (48.3, 95% CI 39.9–56.6), and Eswatini (47.4, 95% CI 35.8–57.1; Supplementary Table S6). Of the 50 highest PAF-ranking countries, the top 10 were in East and Southern Africa, and 14 were in West and Central Africa (Figure 3). In these 50 countries, NHL was the main contributor to the HIV-attributable PAF in 26 countries, KS in 18, and cervical cancer in six (Figure 3).

### HIV-attributable and all-cause ASIR of ADCs

Globally, the overall ASIR of ADCs was 15.7 per 100,000 people (14.1 for cervical cancer, 5.6 for NHL, and 0.4 for KS). ASIRs exceeded 50 per 100,000 people in Eswatini, Zambia, Zimbabwe, Malawi, Uganda, and Mozambique and were lowest in Yemen (<6.0; Figure 2B; Supplementary Table S7). The global ASIR of HIV-attributable ADCs was 1.3 per 100,000 people (0.4 for cervical cancer, 0.6 for NHL, and 0.2 for KS). The highest (>25 per 100,000) HIV-attributable ASIRs were reported in Eswatini (34.9%), Zambia (32.0%), and Mozambique (25.9%), while the lowest (<0.02 per 100,000) were in the Syrian Arab Republic and Bangladesh (Figure 2C; Supplementary Table S7). Among the 50 countries with the highest HIV-attributable ASIR, 18 were in West and Central Africa and 15 in East and Southern Africa. In contrast, the highest all-cause ASIR of ADCs was observed in 16 countries in West and Central Africa and 15 in East and Southern Africa (Supplementary Figure S9).

### Classification of ADC burden

According to all-cause ASIR and HIV-attributable PAF for ADCs, 35 countries were classified as high cancer burden with high HIV attribution, 42 as high cancer burden with low HIV attribution, five as low cancer burden with high HIV attribution, and 59 as low cancer burden and low HIV attribution (Supplementary Table S7). Most countries with high cancer burden and high HIV attribution were in Africa, except for North Africa, while those with low cancer burden and low HIV attribution were mainly in the Middle East, North Africa, Eastern Europe, and Central Asia (Figure 2D).

### Association between PAF, all-cause and HIV-attributable ASIR of ADCs, and HDI

**Figure 4:**
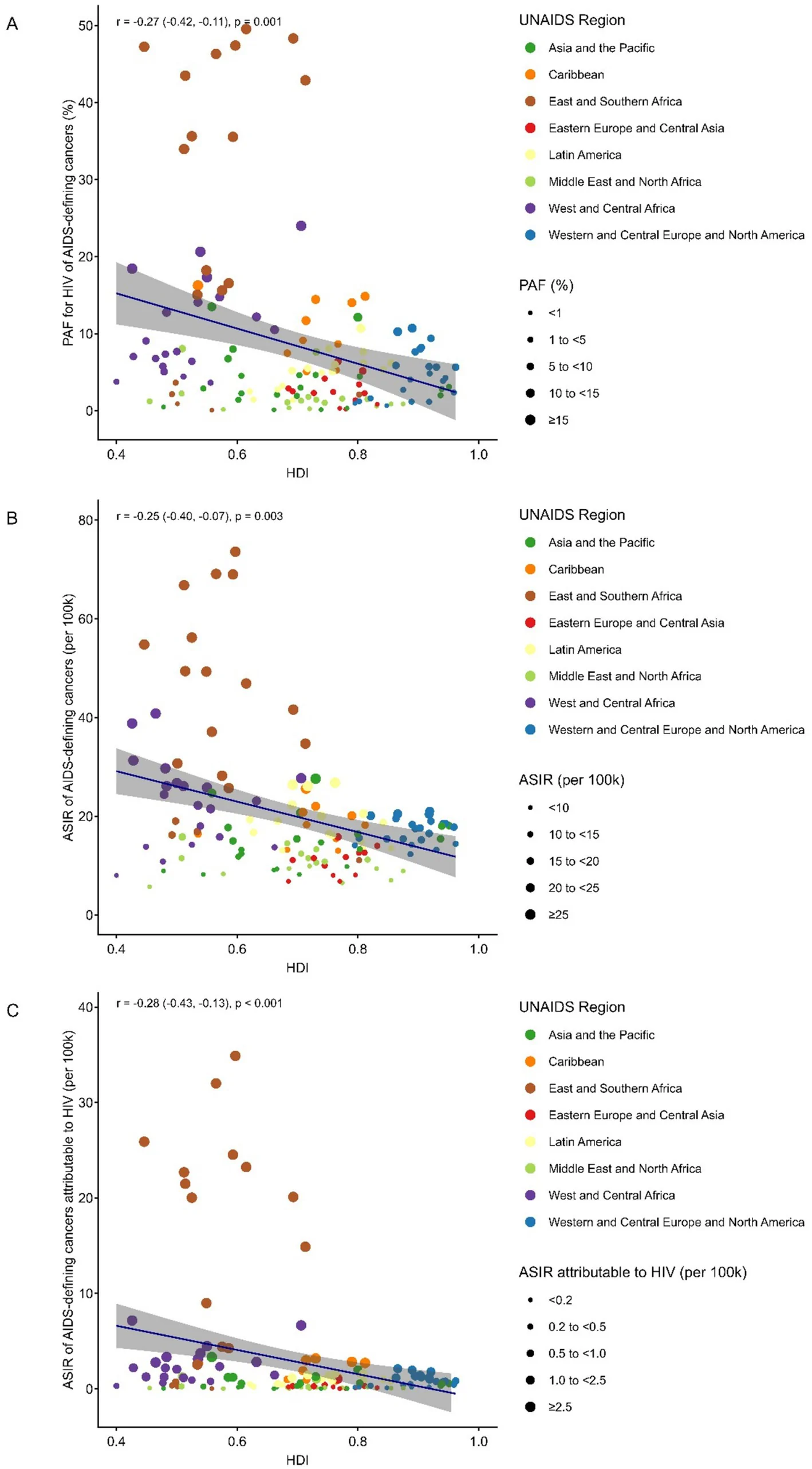
The relationship between PAF, HIV-attributable and all-cause ASIR of ADCs, and HDI. **(A)** The relationship between the PAF of HIV for ADCs and HDI. **(B)** The relationship between ASIR of ADCs and HDI. **(C)** The relationship between the ASIR of ADCs attributable to HIV and HDI. PAF, population attributable fraction. ASIR, age-standardized incidence rate. ADCs, AIDS-defining cancers. HDI, human development index. HIV, human immunodeficiency virus.

At the national level, PAF, all-cause ASIR, and HIV-attributable ASIR of ADCs in PLHIV showed a similar association with HDI. A significant negative relationship was observed between the PAFs of ADCs and HDI (*r* = −0.27, 95% CI −0.42, −0.11, *p* = 0.001; Figure 4A), between all-cause ASIR of ADCs and HDI (*r* = −0.25, 95% CI −0.40 to −0.07, *p* = 0.003; Figure 4B), and between HIV-attributable ASIR of ADCs and HDI (*r* = −0.28, 95% CI −0.43, −0.13, *p* = 0.001; Figure 4C).

## Discussion

Our study provides the first global pooled estimates of HIV-attributable and all-cause ADC burdens, revealing significant disparities across regions, countries, and specific cancers. Globally, over 8% of all-cause ADCs were attributable to HIV infection, with East and Southern Africa bearing the heaviest burden (27.8%) of ADCs linked to HIV. Profound disparities in HIV-attributable ADC burden across UNAIDS regions and PAFs, ranging from under 5% in low-HIV-prevalence regions to over 25% in high-burden areas, reflect global inequalities in HIV epidemiology, cancer biology, and healthcare infrastructure. A recent global assessment estimated that 81,300 of 19 million cancer cases worldwide in 2022 were attributable to HIV, with approximately 70% occurring in Africa and cervical cancer and Kaposi sarcoma constituting the majority (26). These findings corroborate our pooled ADC burden estimates and further underscore that sub-Saharan Africa continues to bear a disproportionate burden of HIV-driven malignancies, even amid expanding ART coverage. Since the United Nations Member States adopted the “95-95-95” targets in June 2021 to close gaps in HIV treatment coverage and outcomes across all sub-populations, age groups, and geographic settings (27), the heavy HIV-attributable ADC burden highlights the need for sustained efforts, particularly in East and Southern Africa, where both HIV and ADC incidence remain high.

This study revealed significant disparities in cancer-specific incidence proportions between all-cause and HIV-attributable ADCs, underscoring HIV’s distinct oncogenic influence across malignancies and regions. While cervical cancer leads all-cause ADCs globally, NHL predominates among HIV-attributable ADCs, reflecting heightened susceptibility of PLHIV to lymphoproliferative malignancies due to immunosuppression and co-infections with oncogenic viruses such as EB virus and HTLV-I (28–30). In contrast, cervical cancer’s dominance among all-cause ADCs aligns with its high global incidence in women, driven primarily by persistent HPV infection (31). These distinctions underscore the dual burden of HIV and other oncogenic factors in different populations and settings, informing the need for tailored public health strategies and clinical interventions.

Regionally, cervical cancer’s predominance among all-cause ADCs in most UNAIDS regions reflects the pervasive burden of HPV-related malignancies, particularly in resource-limited settings (32, 33). Widespread HPV prevalence, coupled with limited access to screening and vaccination in less-developed areas, contributes to its significant role in all-cause ADCs. This emphasizes the critical need to expand HPV vaccination and screening, particularly in LMICs where cervical cancer burden is high (14). The WHO Global Strategy to Eliminate Cervical Cancer, adopted by all 194 Member States in 2020, set ambitious 90–70–90 targets to be achieved by 2030 (34). However, there are significant disparities of HPV vaccination and cervical cancer screening across different areas. HPV vaccination coverage during 2023–2024 averaged 70% in high-income countries (HICs) but only 38% in LMICs (35). Nearly 84% of women aged 30–49 years in HICs had ever been screened in their lifetime, whereas only 27% in LMICs, with the pooled screening proportion of only 10.3% in sub-Saharan Africa (36, 37). Similarly, in Eastern Europe and Central Asia, another region with elevated cervical cancer burden, screening systems remain fragmented, with opportunistic screening and non-standard practices prevailing over organized population-based programs (38). These disparities highlight that the elevated cervical cancer burden in certain regions is driven not only by HIV-associated immunosuppression but also by gaps in access to vaccination and screening. A previous study indicated that the integration of HPV-based screening and screen-and-treat approaches into routine HIV care has been identified as a priority, yet a facility-based survey across sub-Saharan Africa found that only 33% of HIV clinic sites offer cervical screening (39). In fact, besides vaccination and cancer screening, eliminating cervical cancer is also determined by factors of local health systems and socioeconomic level. Therefore, a comprehensive approach is urgently needed to accelerate cervical cancer elimination, encompassing HPV vaccination for adolescent girls, the expansion of HPV-based screening linked to immediate treatment, and the integration of cervical cancer services into existing HIV programs, especially in LMICs.

In Western and Central Europe and North America, NHL’s leading role in all-cause ADCs may relate to a higher prevalence of sedentary lifestyles and obesity (40, 41), which are established NHL risk factors (42, 43). In the Middle East and North Africa, NHL predominance may be linked to high rates of Hepatitis C virus (HCV), *Helicobacter pylori*, and childhood EBV infection (44–46). For HIV-attributable ADCs, NHL’s predominance across most regions underscores the universal impact of HIV-associated immunosuppression on lymphoma risk. NHL was the leading contributor to the PAF of HIV-attributable ADCs in 26 countries. PLHIV are more vulnerable to co-infections with oncogenic viruses such as HTLV-I and EBV (47), which persist and proliferate under immunosuppression, contributing to the pathogenesis of NHL (17). In addition, CD4 cell count <50 cells/μL and cumulative viremia with HIV RNA > 100,000 copies/mL can significantly increase NHL risk by 14.8- and 2.9-fold, respectively (48). Delayed or absent ART initiation, suboptimal adherence, and treatment interruption all contribute to sustained HIV replication and CD4 + depletion, directly raising ADC risk. Moreover, even with viral suppression, incomplete immune reconstitution keeps PLHIV at elevated cancer risk (49). In high-HIV-prevalence regions, controlling immunosuppression through ART and preventing co-infections could help reduce NHL incidence. Given the central role of EBV in the pathogenesis of HIV-associated NHL, and in the absence of effective screening modalities, the development of an effective EBV vaccine represents a potential and promising approach to reduce the lymphoma burden among PLHIV. Although no EBV vaccine has yet received regulatory approval, several candidates currently under development have demonstrated robust immunogenicity and protective efficacy in animal models, underscoring the need for continued and increased investment in this area (50, 51). However, the exceptional prominence of KS in East and Southern Africa and West and Central Africa reflects the co-endemicity of KSHV and HIV in the population (41), highlighting the region-specific role of KSHV co-infection in PLHIV and synergising with HIV to drive the disproportionate KS burden. This underscores the need for integrated HIV-KSHV management strategies tailored to these regions.

Despite substantial reductions in ADC incidence since the widespread rollout of ART, PLHIV continue to experience markedly elevated cancer risks compared with the general population even after prolonged viral suppression. Contemporary evidence indicates that KS remains approximately 800-fold more common and NHL roughly 10-fold more common among PLHIV receiving long-term ART, underscoring the persistent oncogenic consequences of HIV-associated immunosuppression and viral co-infections (52). These residual risks highlight the limitations of ART alone in fully mitigating the HIV-attributable cancer burden and reinforce the need for concurrent oncogenic virus monitoring and early cancer detection strategies. Notably, beyond chronic immunosuppression facilitating oncogenic virus replication, emerging evidence suggests that persistent HIV reservoirs—even under suppressive antiretroviral therapy—may contribute to lymphoma development through provirus integration, viral protein-mediated disturbances, and microenvironment dysregulation (53). Therefore, there is an urgent need to develop accessible strategies that can both eradicate HIV reservoirs and mitigate lymphoma risk.

Our results show a complex interplay between ADC burden and HIV attribution across regions and countries. Nearly one-third of countries face both high cancer burden and high HIV attribution, requiring integrated investments in HIV treatment, prevention, and cancer care, particularly in East and Southern Africa, where the dual burden is most pronounced (54). In contrast, countries with low burden for both may allocate resources toward other health priorities. For countries with a low cancer burden and high HIV attribution, strengthening HIV prevention through promoting safe sex practices, increasing ART access, and reducing stigma associated with HIV is critical (55). In countries with high cancer burden but low HIV attribution, broader cancer prevention strategies, like tobacco control, vaccination programs (HPV and hepatitis B), and healthy lifestyle promotion, should be prioritized (56). Further research is needed to understand the socioeconomic drivers of cancer and HIV disparities. Policymakers should tailor comprehensive health policies to national needs, supported by international collaborations and shared best practices and resources.

Our findings revealed that HDI was negatively associated with PAF, all-cause ASIR, and HIV-attributable ASIR of ADCs among PLHIV at the country level. Several factors may explain these correlations. First, less-developed countries often face a more severe HIV/AIDS epidemic than developed countries (57), which can increase ADC risk through immunosuppression and viral co-infections. Second, better ART access in higher-HDI countries reduces AIDS progression and ADC incidence (54). Third, higher-HDI countries often have more comprehensive cancer screening programs, leading to early detection and treatment of cancers (58). Finally, improved healthcare infrastructure and lifestyle factors in these countries contribute to lower cancer incidence (59). Given the stronger influence of HDI on HIV-attributable ADC burden, targeted interventions in low-HDI countries could yield significant public health benefits. This may involve improving healthcare infrastructure, increasing access to HIV treatment, promoting cancer screening, and addressing socioeconomic disparities to mitigate the global burden of HIV-related cancers.

The expansion of universal ART coverage holds substantial promise for further reducing the global burden of HIV-attributable ADCs, yet the incidence of ADCs in the ART era remains much higher than in HIV-negative populations, and long-term viral suppression does not fully eliminate excess cancer risk (60). Therefore, ART alone is insufficient to fully mitigate the HIV-attributable ADC burden. Furthermore, as PLHIV age on ART, the burden of non-AIDS-defining cancers will increase, necessitating expanded screening and prevention beyond traditional ADCs. Future progress will depend not only on scaling ART to achieve the UNAIDS 95–95-95 targets, but also on strengthening health systems to deliver integrated HIV-cancer care, implementing evidence-based screening protocols tailored to PLHIV in low-resource settings, and addressing persistent co-infections with oncogenic viruses. Such a comprehensive approach offers the optimal pathway to substantially reduce the dual burden of HIV and cancer globally.

## Limitations

The results of this study should be interpreted with caution due to certain limitations. First, we treated OR, RR, HR, IRR, and SIR equivalently when calculating pooled RRs, as these values were similar in this context. The potential heterogeneity across these measures should be considered cautiously. This meta-analysis included studies with varying methodological quality. Some studies with a higher risk of bias could have influenced the pooled RR and PAF estimates. Second, the exclusion of 31 countries owing to missing HIV prevalence data may introduce regional biases and underestimate the global burden of HIV-attributable ADCs. Third, our uncertainty analysis did not account for the inherent variability in GLOBOCAN estimates, which rely on diverse data sources and modeling assumptions. In addition, cross-country differences in cancer registration quality and reporting practices—particularly in regions with limited healthcare infrastructure—may introduce additional imprecision (61). Fourth, although the method used to estimate the PAF in our study has been widely applied, it considers only the effects of HIV prevalence and RR values, and not the complex interactions between HIV infection and other oncogenic viral infections, or host factors. Fifth, classifying countries into high- or low-burden categories based on global averages may obscure the intricate heterogeneity within nations, particularly in geographically expansive or socioeconomically diverse countries. Relying on global averages can undermine the development of targeted public health interventions, leading to inefficient resource allocation and exacerbating health inequalities (62). Finally, the study used the HDI as a proxy for socioeconomic status and healthcare infrastructure. Although the HDI is a useful composite indicator, it may not capture all the relevant factors that influence the incidence of ADCs, such as specific healthcare policies, cultural practices, and individual behaviors (63).

We estimated HIV-attributable cancer burden using Levin’s PAF formula, which assumes a causal relationship between HIV and each ADC, no confounding, and homogeneous exposure effects across populations. However, these assumptions are unlikely to hold uniformly across diverse global regions, given substantial heterogeneity in HIV epidemiology, ART coverage, prevalence of viral co-infections (e.g., KSHV, EBV, HPV), and healthcare system capacity—all of which may introduce bias into PAF estimates. To improve representativeness, we used region-specific relative risks for PAF calculations. Ideally, risk estimates should be further stratified by both region and time period, but this was not feasible due to insufficient studies across settings. Therefore, our PAF estimates should be interpreted as approximations of the population-level burden of HIV-associated cancers, rather than precise causal effect measures. Future studies with individual-level data from varied settings are warranted to validate and refine these estimates.

We assumed that HIV prevalence among KS cases is equivalent to the PAF, which was employed in previous IARC global burden estimates. However, the assumption may introduce bias (23). Overestimation may occur when the relative risk is finite, particularly in regions where HIV-negative KS, KSHV-driven KS constitutes a meaningful proportion of cases. Conversely, underestimation may arise from HIV under-ascertainment (leading to underestimated HIV prevalence among KS cases), and from the fact that declining RR of KS in the ART era may not fully capture the historical contribution of HIV to KS burden. Therefore, PAF estimates for KS should be interpreted as approximations of the potential population-level burden, and future studies with individual-level data linking HIV status and KS diagnosis are needed to refine these estimates.

## Conclusion

In summary, an estimated 8.1% of global ADC cases were linked to HIV infection in 2022, with East and Southern Africa bearing the highest burden. NHL dominated HIV-attributable ADCs globally, reflecting the synergistic effect of HIV-induced immunosuppression and oncogenic viral co-infections, whereas cervical cancer remained the primary driver of all-cause ADCs due to pervasive HPV prevalence in women. KS was disproportionately concentrated in sub-Saharan Africa, driven by high KSHV co-infection rates. A lower HDI was strongly associated with higher ADCs and HIV-attributable ADC burdens, underscoring the impact of limited healthcare access and ART coverage. These findings highlight the complex interplay between HIV and oncogenic processes, emphasizing the need for a multifaceted approach to address the dual burden of HIV-attributable and all-cause ADCs, particularly in sub-Saharan Africa. By understanding the distinct oncogenic influences of HIV and other factors, public health strategies can be improved to effectively reduce the incidence of these cancers in different populations, including scaling up ART coverage, expanding HPV vaccination, monitoring oncogenic viruses, promoting cervical screening in low-resource settings, and prioritizing NHL or KS prevention in high-HIV regions.

## Data Availability

The original contributions presented in the study are included in the article/Supplementary material, further inquiries can be directed to the corresponding authors.

## Glossary

ADC: AIDS-defining cancer
ART: Antiretroviral therapy
ASIR: Age-standardized incidence
CI: Confidence intervals
EBV: Epstein–Barr virus
HIV: Human immunodeficiency virus
HDI: Human Development Index
HPV: Human papillomavirus
HR: Hazard ratio
HTLV-I: Human T-cell leukemia/lymphoma virus type I
IARC: The International Agency for Research on Cancer
IRR: Incidence rate ratio
KS: Kaposi sarcoma
KSHV: Kaposi sarcoma-associated herpesvirus
LMICS: low- and middle-income countries
NHL: Non-Hodgkin lymphoma
OR: Odds ratio
PAF: Population-attributable fraction
PLHIV: People living with HIV
RR: risk ratio
SIR: Standardized incidence ratio
UNAIDS: The Joint United Nations Program on HIV/AIDS
