# Supplementary material for: Estimates of the global burden of HIV-attributable and all-cause AIDS-defining cancers: a population-based modeling study
Source: Front Public Health. 2026 Sep 10;14:1933709. doi: 10.3389/fpubh.2026.1933709 (PMC13601286; doi:10.3389/fpubh.2026.1933709)
Supplement: Supplementary file 1 [file Data_Sheet_1.doc]

Contents

Appendix 1. Method for calculating country-specific estimation of AIDS-defining cancers attributable to HIV infection	2

Table S1. Characteristics of included studies for cervical cancer	7

Table S2. Characteristics of included studies for non-Hodgkin lymphoma	9

Table S3. Characteristics of included studies for Kaposi sarcoma	12

Figure. S1. Funnel plots for evaluating publication bias in the meta-analyses of the association between HIV infection and the risk of (A) cervical cancer, (B) non-Hodgkin lymphoma, and (C) Kaposi sarcoma	16

Table S4. Subgroup analysis for pooled risk ratio by UNAIDS regions	17

Table S5. Subgroup analysis for pooled risk ratio by UNAIDS regions (excluding studies identified as having higher potential for publication bias)	18

Table S6. Estimated population attributable fraction of HIV for AIDS-defining cancers in 2022, by country	19

Table S7. Estimated age-standardised incidence rate attributable to HIV of AIDS-defining cancers in 2022, by country	32

Appendix 2. Estimated AIDS-defining cancer burden in excluded countries having no HIV prevalence data	39

Figure. S2. Flow chart of the search for cervical cancer and newly added studies	40

Figure. S3. Flow chart of the search for non-Hodgkin lymphoma and newly added studies	41

Figure. S4. Flow chart of the search for Kaposi sarcoma and newly added studies	42

Figure. S5. Forest plot showing the pooled risk ratio of developing cervical cancer in women living with HIV	43

Figure. S6. Forest plot showing the pooled risk ratio of developing non-Hodgkin lymphoma in the population living with HIV	44

Figure. S7. Forest plot showing the pooled risk ratio of developing Kaposi sarcoma in the population living with HIV	45

Figure. S8. Population attributable fraction (PAF) of HIV for AIDS-defining cancers, stratified by UNAIDS regions	46

Figure. S9. Estimated HIV-attributable and all-cause age-standardised incidence rate in the 50 highest-ranked countries	47

Appendix 1. Method for calculating country-specific estimation of AIDS-defining cancers attributable to HIV infection

*Step 1*

We performed a systematic literature review and meta-analysis to calculate the pooled risk ratio for cervical cancer and non-Hodgkin lymphoma associated with HIV infection. We anticipated heterogeneity between studies when estimating the risk ratio due to different study designs, methods of analysis and varying adjustment, and geographical and population differences. We therefore used a random effects model to account for both within and between study heterogeneity. We calculated a pooled RR value for these UNAIDS regions when the number of estimates reaches five. Otherwise, region pooled RR is equal to the corresponding global one.

*Step 2*

We used a random-effects model to account for within and between study heterogeneity. We extracted risk ratios for cervical cancer and non-Hodgkin lymphoma from the relevant studies. The risk ratios were then pooled using a random effects model in the meta for package in R.

*Step 3*

We obtained age-specific HIV prevalence rates in 2022 for 173 countries from the online website of UNAIDS. Similarly, we obtained age-specific estimates of cervical cancer, non-Hodgkin lymphoma and Kaposi sarcoma cases in 185 countries from GLOBOCAN 2022 provided by the International Agency for Research on Cancer (IARC). Through combing these two data sources, the statistical analysis of latest global burden of AIDS-defining cancers in 2022 was available in 142 countries.

*Step 4*

Assumptions:

• We assumed that the pooled risk ratios obtained from a range of registry linkage, cohort and case-control studies were applicable across both low- and middle-income, and high-income countries.

• The degree of adjustment for confounders varied between the primary studies and therefore estimates maybe limited by residual confounding.

• No correlation within studies was taken into account (for studies with more than one estimate); the estimates were considered to be independent.

*Step 5*

We estimated the population attributable fraction for cervical cancer and non-Hodgkin lymphoma as follows:

Population Attributable Fraction (PAF) =

The prevalence of HIV infection in Kaposi sarcoma cases was assumed to equate with PAF. We conduct a meta-analysis for calculating the population attributable fraction (PAF) of Kaposi sarcoma based on UNAIDS region, when the number of estimates reaches five, otherwise, region pooled RR is equal to the corresponding global one. Then, the PAF values are subsequently allocated to specific countries within each region.

*Step 6*

Multiplying the PAF with the total number of cervical cancer, non-Hodgkin lymphoma and Kaposi sarcoma cases yielded the total number of cervical cancer, non-Hodgkin lymphoma and Kaposi sarcoma cases attributable to HIV, respectively.

Ncases attributable to HIV = *PAF* * Ncases

*Step 7*

The total number of AIDS-defining cancers is calculated by summing the cases of cervical cancer, non-Hodgkin lymphoma, and Kaposi sarcoma. The total number of HIV-attributable AIDS-defining cancers is obtained by summing the HIV-attributable cases of these three malignancies. The population attributable fraction (PAF) for AIDS-defining cancers is then derived by dividing the total HIV-attributable cases by the total number of AIDS-defining cancer cases.

Ncases of AIDS-defining cancers = Ncases of cervical cancer + Ncases of non-Hodgkin lymphoma + Ncases of Kaposi sarcoma

Ncases of HIV-attributable AIDS-defining cancers = Ncases of HIV-attributable cervical cancer + Ncases of HIV-attributable non-Hodgkin lymphoma + Ncases of HIV-attributable Kaposi sarcoma

PAFAIDS-defining cancers = Ncases of HIV-attributable AIDS-defining cancers / Ncases of AIDS-defining cancers

*Step 8*

We estimated the uncertainty intervals for AIDS-defining cancer cases associated with HIV infection using simulation. We sampled from the probability distributions which captured the uncertainty around the estimate for each component (specifically, log normal for rate ratios, normal for the cervical cancer numbers and beta for the prevalence of HIV infection) calculating the PAF, proportion and numbers for each of 10,000 samples. Total numbers for each country were reported with uncertainty estimates as 95% confidence interval. The uncertainty ranges can be viewed as approximate Bayesian posterior probability limits with very diffuse priors for the estimates and risk factor rate ratios.

Example for Zimbabwe:

The HIV prevalence is represented as 10,000 samples of a variable following a beta distribution (left upper figure). The pooled risk ratio relative risk estimator of interest is presented in the bottom left figure as a histogram of 10 000 samples of a normally distributed variable. Using the above-mentioned formula, we simulated 10,000 samples of the PAF.

We calculated the population attributable fraction (PAF) for non-Hodgkin lymphoma using the same methodology.

In the second step, we simulated the number of cervical cancer cases for Zimbabwe (bottom left figure) and multiplied it with the simulated PAF for Zimbabwe (left upper figure). The result was the number of cervical cancer cases attributable to HIV for Zimbabwe (right figure).

We calculated the the number of non-Hodgkin lymphoma and Kaposi sarcoma cases using the same methodology.

In the third step, we simulated the number of AIDS-defining cancer cases for Zimbabwe (right figure) by summing the cases of cervical cancer (left upper figure), non-Hodgkin lymphoma (left middle figure), and Kaposi sarcoma (left bottom figure).

In the forth step, we simulated the total number of HIV-attributable AIDS-defining cancers (right figure) by summing the HIV-attributable cases of these three malignancies (left figure).

In the fifth step, the population attributable fraction (PAF) for AIDS-defining cancers is derived by dividing the total HIV-attributable cases by the total number of AIDS-defining cancer cases.

We ran these analyses for all countries and integrated the results into regional and global estimates.

Table S1. Characteristics of included studies for cervical cancer

| Author and year | Study period | Country/region | UNAIDS region | Study type | Number of participants | Risk measure | Risk value | Risk of bias |
| --- | --- | --- | --- | --- | --- | --- | --- | --- |
| Cooksley et al, 1999 | 1981–1994 | USA | Western and Central Europe and North America | Registry linkage study | 14986 | SIR | 2.5 | High |
| Serraino et al, 1999 | 1988–1998 | Italy/France | Western and Central Europe and North America | Registry linkage study | 2952 | SIR | 2.8 | Middle |
| Phelps et al, 2001 | 1993–1995 | USA | Western and Central Europe and North America | Cohort study | 1310 | IRR | 7.99 | Middle |
| Clifford et al, 2005 | 1985–2003 | Switzerland | Western and Central Europe and North America | Cohort study | 7304 | SIR | 8 | Middle |
| Mbulaiteye et al, 2006 | 1988–2002 | Uganda | East and Southern Africa | Registry linkage study | 12607 | SIR | 2.7 | High |
| Sekirime et al, 2007 | 1993–1995 | Uganda | East and Southern Africa | Case-control study | 232 | OR | 1.26 | High |
| Kahesa et al, 2008 | 2007–2007 | Tanzania | East and Southern Africa | Case-control study | 276 | OR | 2.9 | High |
| Bedimo et al, 2009 | 1997–2004 | USA | Western and Central Europe and North America | Cohort study | 100260 | IRR | 12.8 | Low |
| Holmes et al, 2009 | 1998–2000 | Senegal | West and Central Africa | Case-control study | 5929 | OR | 6.5 | Middle |
| Adjorlolo et al, 2010 | 1997–1999 | Côte d'Ivoire | West and Central Africa | Case-control study | 252 | OR | 1.8 | Middle |
| Vogel et al, 2011 | 1996–2009 | Germany | Western and Central Europe and North America | Registry linkage study | 1476 | SIR | 4 | Middle |
| Zhang et al, 2011 | 2004–2008 | China | Asia and the Pacific | Registry linkage study | 3554 | SIR | 68.1 | Middle |
| Tanon et al, 2012 | 2009–2011 | Côte d'Ivoire/Benin | West and Central Africa | Cohort study | 1017 | OR | 7.9 | Middle |
| Abraham et al, 2013 | 1996–2010 | USA | Western and Central Europe and North America | Cohort study | 25711 | SIR | 4.1 | Low |
| Akarolo et al, 2014 | 2005–2012 | Nigeria | West and Central Africa | Cohort study | 17826 | SIR | 2 | High |
| Chen et al, 2014 | 2000–2008 | China | Asia and the Pacific | Cohort study | 359501 | SIR | 4.1 | Middle |
| Jaquet et al, 2015 | 2009–2012 | Benin/ Côte d'Ivoire/ Nigeria/Togo | West and Central Africa | Case-control study | 1644 | OR | 4.3 | Middle |
| Godbole et al, 2016 | 1991–2009 | India | Asia and the Pacific | Registry linkage study | 32575 | SIR | 15.7 | Middle |
| Mayor et al, 2016 | 1991–2009 | Puerto Rico | Caribbean | Registry linkage study | 132 | SIR | 47 | High |
| Salters et al, 2016 | 1994–2008 | Canada | Western and Central Europe and North America | Registry linkage study | 2211 | SIR | 24.18 | Middle |
| Hernández et al, 2017 | 1996–2012 | USA | Western and Central Europe and North America | Registry linkage study | 129995 | SIR | 3.24 | Middle |
| Massad et al, 2017 | 1994–2015 | USA | Western and Central Europe and North America | Registry linkage study | 2295 | SIR | 3.31 | Middle |
| Mpunga et al, 2018 | 2012–2016 | Rwanda | East and Southern Africa | Case-control study | 3852 | OR | 5.9 | Middle |
| Grabar et al, 2019 | 1992–2009 | France | Western and Central Europe and North America | Registry linkage study | 28977 | SIR | 5.4 | Middle |
| Dhokotera et al, 2019* | 2004-2014 | South African | West and Central Africa | Case-control study | 329208 | AOR | 1.70 | Middle |
| Liping et al, 2020* | 2004-2018 | China | Asia and the Pacific | Cohort study | 22980 | SIR | 5.22 | Middle |
| Michaud et al, 2021* | 2001-2012 | USA | Western and Central Europe and North America | Cohort study | 443592 | aHR | 3.27 | Middle |
| Soon et al, 2022* | 2002-2017 | South Korea | Asia and the Pacific | Cohort study | 11737 | SIR | 7.5 | Middle |
| Stier et al, 2022* | 2002-2016 | USA | Western and Central Europe and North America | Cohort study | 164084 | SIR | 3.4 | Middle |
| Teresa et al, 2022* | 2010–2015 | France | Western and Central Europe and North America | Cohort study | 44642 | SIR | 1.93 | Middle |
| Boyoung Park et al, 2022* | 2006-2018 | Korea | Asia and the Pacific | Cohort study | 11552 | SIR | 4.98 | Middle |
| Hele et al, 2022* | 2010-2017 | France | Western and Central Europe and North America | Cohort study | 3572 | OR | 7.60 | High |
| Zhou et al, 2023* | 2012 | USA | Western and Central Europe and North America | Cross-sectional study | 17426471 | OR | 5.34 | Middle |
| Senga et al, 2023* | 1995-2016 | South African | East and Southern Africa | Case-control study | 9559 | AOR | 2.7 | Low |
| Fern ´Andez Villalobos et al, 2024* | 2011-2020 | South African | East and Southern Africa | Cohort study | 517312 | aHR | 2.99 | Middle |
| Tisler et al, 2024* | 2004-2021 | Estonia | Western and Central Europe and North America | Cohort study | 7011 | SIR | 5.8 | Middle |
| Dusiingize et al, 2024* | 2007-2018 | Rwanda | East and Southern Africa | Case-control study | 17679 | OR | 2.31 | Middle |

* Newly added studies based on the previously published literature review.

Table S2. Characteristics of included studies for non-Hodgkin lymphoma

| Author and year | Study period | Country/region | UNAIDS region | Study type | Number of participants | Risk measure | Risk value | Risk of bias |
| --- | --- | --- | --- | --- | --- | --- | --- | --- |
| Sitas et al, 1997 | 1992–1995 | South Africa | East and Southern Africa | Case-control study | 1238 | OR | 4.8 | Middle |
| Serraino et al, 1997 | 1980–1995 | Italy | Western and Central Europe and North America | Cohort study | 1255 | SIR | 157.3 | Middle |
| Franceschil et al, 1998 | 1976–1994 | Italy | Western and Central Europe and North America | Registry linkage study | 6067 | SIR | 77.1 | Middle |
| Cooksley et al, 1999 | 1985–1994 | USA | Western and Central Europe and North America | Cohort study | 2289 | SIR | 33.7 | Low |
| Petruckevitch et al, 1999 | 1982–1995 | UK | Western and Central Europe and North America | Registry linkage study | 992 | SIR | 15.0 | Middle |
| Grulich et al, 1999 | 1980–1993 | Australia | Asia and the Pacific | Cohort study | 3616 | SIR | 128.0 | Middle |
| Sitas et al , 2000 | 1995–1999 | South Africa | East and Southern Africa | Case-control study | 5727 | OR | 5.0 | Middle |
| Gallagher et al, 2001 | 1981–1994 | USA | Western and Central Europe and North America | Registry linkage study | 10083 | SIR | 54.6 | Middle |
| Gallagher et al, 2001 | 1981–1994 | USA | Western and Central Europe and North America | Registry linkage study | 1288 | SIR | 37.4 | Middle |
| Frisch et al, 2001 | 1978–1996 | USA | Western and Central Europe and North America | Registry linkage study | 302834 | SIR | 72.8 | Low |
| Wilde et al, 2002 | 1978–1999 | UK | Western and Central Europe and North America | Cohort study | 89 | SIR | 76.1 | High |
| Frisch et al, 2003 | 1989–1997 | Denmark | Western and Central Europe and North America | Cohort study | 5005 | SIR | 19.9 | Middle |
| Hessol et al, 2004 | 1994–2001 | USA | Western and Central Europe and North America | Cohort study | 1950 | SIR | 19.0 | Middle |
| Newnham et al, 2005 | 1985–2001 | UK | Western and Central Europe and North America | Registry linkage study | 33190 | SIR | 9.5 | Middle |
| Busnach et al, 2006 | 1970–2005 | France/Italy | Western and Central Europe and North America | Cohort study | 8074 | SIR | 81.7 | Middle |
| Mbulaiteye et al, 2006 | 1988–2002 | Uganda | East and Southern Africa | Registry linkage study | 12607 | SIR | 4.7 | Middle |
| Engels et al, 2006 | 1996–2002 | USA | Western and Central Europe and North America | Registry linkage study | 107417 | SIR | 22.6 | Middle |
| Galceran et al, 2007 | 1981–1999 | Spain | Western and Central Europe and North America | Registry linkage study | 1304 | SIR | 126.1 | Middle |
| Srisawat et al, 2008 | 1997–2007 | Thailand | Asia and the Pacific | Cohort study | 863 | SIR | 33.2 | Middle |
| Dhir et al, 2008 | 2001–2005 | India | Asia and the Pacific | Cohort study | 166 | SIR | 10.3 | Middle |
| Dhir et al, 2008 | 2001–2005 | India | Asia and the Pacific | Cohort study | 85 | SIR | 17.1 | Middle |
| van Leeuwen et al, 2009 | 1982–1995 | Australia | Asia and the Pacific | Cohort study | 20232 | SIR | 75.7 | Low |
| van Leeuwen et al, 2009 | 1996–1999 | Australia | Asia and the Pacific | Cohort study | 20232 | SIR | 36.9 | Low |
| van Leeuwen et al, 2009 | 2000–2004 | Australia | Asia and the Pacific | Cohort study | 20232 | SIR | 12.7 | Low |
| Silverberg et al, 2009 | 1996–2007 | USA | Western and Central Europe and North America | Cohort study | 222590 | RR | 18.1 | Middle |
| Dal Maso et al, 2009 | 1997–2004 | Italy | Western and Central Europe and North America | Registry linkage study | 21951 | SIR | 93.4 | Low |
| Seaberg et al, 2010 | 1984–2007 | USA | Western and Central Europe and North America | Cohort study | 6949 | SIR | 36.8 | Low |
| Vajdic et al, 2010 | 1982–2004 | Australia | Asia and the Pacific | Cohort study | 17175 | SIR | 34.7 | Middle |
| Lanoy et al, 2010 | 1987–2002 | USA | Western and Central Europe and North America | Case-control study | 149630 | OR | 2.4 | Middle |
| Franceschi et al, 2010 | 1985–1996 | Swiss | Western and Central Europe and North America | Registry linkage study | 9429 | SIR | 135.5 | Middle |
| Franceschi et al, 2010 | 1997–2001 | Swiss | Western and Central Europe and North America | Registry linkage study | 9429 | SIR | 35.1 | Middle |
| Franceschi et al, 2010 | 2002–2006 | Swiss | Western and Central Europe and North America | Registry linkage study | 9429 | SIR | 21.3 | Middle |
| Zhang et al, 2011 | 2004–2008 | China | Asia and the Pacific | Cohort study | 3554 | SIR | 34.5 | Middle |
| Tanon et al, 2012 | 2009–2011 | Côte d’Ivoire/Benin | West and Central Africa | Case-control study | 1459 | OR | 4.0 | Middle |
| Hleyhel et al, 2013 | 1992–1996 | France | Western and Central Europe and North America | Cohort study | 99309 | SIR | 116.7 | Middle |
| Hleyhel et al, 2013 | 1997–2000 | France | Western and Central Europe and North America | Cohort study | 99309 | SIR | 33.6 | Middle |
| Hleyhel et al, 2013 | 2001–2004 | France | Western and Central Europe and North America | Cohort study | 99309 | SIR | 15.4 | Middle |
| Hleyhel et al, 2013 | 2005–2009 | France | Western and Central Europe and North America | Cohort study | 99309 | SIR | 9.1 | Middle |
| Chen et al, 2014 | 1998–2009 | China | Asia and the Pacific | Cohort study | 15269 | SIR | 26.1 | Low |
| Jaquet et al, 2015 | 2009–2012 | Côte d’Ivoire/Benin/ Nigeria/Togo | West and Central Africa | Case-control study | 2436 | OR | 3.6 | Middle |
| Salters et al, 2016 | 1994–2008 | Canada | Western and Central Europe and North America | Cohort study | 2211 | SIR | 14.5 | Middle |
| Godbole et al, 2016 | 1996–2008 | India | Asia and the Pacific | Registry linkage study | 613 | SIR | 14.0 | Middle |
| Lee et al, 2016 | 2006–2012 | USA | Western and Central Europe and North America | Cohort study | 63221 | SIR | 4.2 | Middle |
| Hernández et al, 2017 | 1996–2012 | USA | Western and Central Europe and North America | Registry linkage study | 448258 | SIR | 11.5 | Middle |
| Tanaka et al, 2017 | 1997–2012 | Brazil | Latin America | Registry linkage study | 1461 | SIR | 14.1 | Middle |
| Tanaka et al, 2017 | 1997–2012 | Brazil | Latin America | Registry linkage study | 539 | SIR | 13.5 | Middle |
| Hessol et al, 2018 | 1985–2013 | USA | Western and Central Europe and North America | Registry linkage study | 22623 | SIR | 17.2 | Middle |
| Park et al, 2022 | 2006–2018 | South Korea | Asia and the Pacific | Cohort study | 10444 | SIR | 11.8 | Middle |
| Park et al, 2022 | 2006–2018 | South Korea | Asia and the Pacific | Cohort study | 1108 | SIR | 15.6 | Middle |
| Lee et al, 2022 | 2004–2017 | South Korea | Asia and the Pacific | Cohort study | 11737 | SIR | 21.7 | Middle |
| Sengayi et al, 2023* | 1995-2016 | South Africa | East and Southern Africa | Case-control study | 10326 | OR | 11.3 | Middle |
| Zhou et al, 2023* | 2012 | USA | Western and Central Europe and North America | Cross-sectional study | 17353963 | OR | 30.6 | Middle |
| Dusingize et al, 2024* | 2007-2018 | Rwanda | East and Southern Africa | Cohort study | 5813 | OR | 1.6 | Middle |
| Lee et al, 2024* | 2000-2016 | USA | Western and Central Europe and North America | Cohort study | 387767 | RR | 6.0 | Middle |
| McGee-Avila et al, 2024* | 2001-2019 | USA | Western and Central Europe and North America | Registry linkage study | 2880955 | OR | 9.0 | Middle |

* Newly added studies based on the previously published literature review.

Table S3. Characteristics of included studies for Kaposi sarcoma

| Author and year | Study period | Country/  region | UNAIDS region | Localization | Number of KS cases | Number of HIV cases | HIV prevalence/  incidence (%) | Risk of bias |
| --- | --- | --- | --- | --- | --- | --- | --- | --- |
| Noel et al, 1996 | 1996 | Belgium | Western and Central Europe and North America | All site | 78 | 61 | 78.2 | Middle |
| Newton. et al, 2001 | 1994-1998 | Uganda | East and Southern Africa | All site | 36 | 29 | 80.6 | Middle |
| Hsu et al, 2001 | 1993-1999 | China | Asia and the Pacific | All site | 15 | 2 | 13.3 | High |
| Hong. et al, 2002 | 1990-1999 | Australia | Asia and the Pacific | All site | 44 | 33 | 75.0 | Middle |
| Guttman et al, 2003 | 1980-1998 | Israel | Western and Central Europe and North America | All site | 1630 | 60 | 3.7 | Low |
| Marcelin et al, 2004 | 2000-2001 | France | Western and Central Europe and North America | Oral KS | 28 | 17 | 60.7 | High |
| Mohammed et al, 2005 | 1994-1998 | Nigeria | West and Central Africa | All site | 32 | 19 | 59.4 | Middle |
| Dal et al, 2005 | 1985-1998 | Italy | Western and Central Europe and North America | All site | 1508 | 633 | 42.0 | Low |
| Mseddi et al, 2005 | 1981-2003 | Tunisia | Middle East and North Africa | All site | 65 | 1 | 1.5 | Middle |
| Hbid et al, 2005 | 2002-2003 | Morocco | Middle East and North Africa | All site | 26 | 8 | 30.8 | High |
| Mwakigonja et al, 2007 | 1990-2005 | United Republic of Tanzania | East and Southern Africa | All site | 78 | 74 | 94.9 | Middle |
| Ramos-da-Silva et al, 2007 | 1986-2002 | Brazil | Latin America | All site | 47 | 24 | 52.1 | Middle |
| Stein et al, 2008 | 1995-2004 | South Africa | East and Southern Africa | All site | 333 | 297 | 89.2 | Low |
| Mbah et al, 2008 | 1994-2004 | Nigeria | West and Central Africa | All site | 27 | 16 | 59.3 | High |
| Asuquo et al, 2008 | 2005-2006 | Nigeria | West and Central Africa | All site | 11 | 9 | 81.8 | High |
| Robey et al, 2009 | 2009 | United Kingdom | Western and Central Europe and North America | All site | 14 | 12 | 85.7 | High |
| Caterino et al, 2010 | 2008 | Mozambique | East and Southern Africa | All site | 24 | 17 | 70.8 | High |
| Roiz et al, 2010 | 2005-2006 | Zimbabwe | East and Southern Africa | All site | 702 | 669 | 95.3 | Low |
| Dogan et al, 2010 | 1998-2009 | Turkey | Western and Central Europe and North America | All site | 51 | 1 | 2.0 | Middle |
| Shiels et al, 2011 | 1996-2007 | USA | Western and Central Europe and North America | All site | 22063 | 16260 | 73.7 | Low |
| Solivetti et al, 2011 | 2010 | Italy | Western and Central Europe and North America | Cutaneous KS | 24 | 8 | 33.3 | High |
| Ben et al, 2011 | 1978-1998 | Tunisia | Middle East and North Africa | All site | 91 | 2 | 2.2 | Middle |
| Tiussi et al, 2012 | 1986-2009 | Brazil | Latin America | All site | 15 | 12 | 80.0 | High |
| Chang et al, 2012 | 1998-2012 | Republic of Korea | Asia and the Pacific | Cutaneous KS | 17 | 1 | 5.9 | High |
| Zaraa et al, 2012 | 1982-2007 | Tunisia | Middle East and North Africa | All site | 75 | 4 | 5.3 | Middle |
| Jalilvand et al, 2012 | 2000-2009 | Iran | Middle East and North Africa | Cutaneous KS | 43 | 2 | 4.7 | Middle |
| Azadmanesh et al, 2012 | 1999-2010 | Iran | Middle East and North Africa | All site | 36 | 2 | 5.6 | Middle |
| Forae et al, 2013 | 1994-2013 | Nigeria | West and Central Africa | All site | 79 | 76 | 96.2 | Middle |
| Laresche et al, 2014 | 1997-2009 | France | Western and Central Europe and North America | All site | 57 | 35 | 61.4 | Middle |
| Wu et al, 2014 | 1997-2013 | China | Asia and the Pacific | All site | 105 | 28 | 26.7 | Low |
| Chalya et al, 2015 | 2004-2014 | United Republic of Tanzania | East and Southern Africa | All site | 248 | 122 | 49.0 | Low |
| Ngalamika et al, 2015 | 2008-2013 | Zambia | East and Southern Africa | All site | 726 | 460 | 63.4 | Low |
| Jaquet. et al, 2015 | 2011-2012 | Nigeria | West and Central Africa | All site | 22 | 11 | 50.0 | High |
| Jaquet et al, 2015 | 2011-2012 | Togo | West and Central Africa | All site | 5 | 3 | 60.0 | High |
| Jaquet. et al, 2015 | 2009-2010 | Côte d'Ivoire | West and Central Africa | All site | 29 | 21 | 72.4 | High |
| Mittermayer et al, 201 | 2002-2014 | Malawi | East and Southern Africa | All site | 191 | 171 | 89.5 | Low |
| Sengai et al, 2017 | 2004-2012 | South Africa | East and Southern Africa | All site | 370 | 357 | 96.5 | Low |
| Host. et al, 2017 | 2014-2016 | Malawi | East and Southern Africa | All site | 174 | 153 | 87.9 | Low |
| Moses. et al, 2017 | 2009-2012 | Malawi | East and Southern Africa | All site | 52 | 49 | 94.2 | Middle |
| Pérez et al, 2017 | 2000-2013 | Argentina | Latin America | All site | 24 | 11 | 45.8 | High |
| Limbau. et al, 2017 | 2010-2014 | Romania | Western and Central Europe and North America | All site | 82 | 15 | 18.3 | Middle |
| Carmo et al, 2017 | 2006-2015 | Portugal | Western and Central Europe and North America | Gastrointestinal KS | 13 | 10 | 76.9 | High |
| Awazawa et al, 2017 | 1984-2014 | Japan | Asia and the Pacific | All site | 74 | 13 | 18.0 | Middle |
| Mpunga et al, 2018 | 2012-2016 | Rwanda | East and Southern Africa | All site | 60 | 49 | 81.7 | Middle |
| Yazici et al, 2018 | 2005-2017 | Turkey | Western and Central Europe and North America | All site | 90 | 1 | 1.1 | Middle |
| Polat et al, 2018 | 2008-2016 | Turkey | Western and Central Europe and North America | All site | 77 | 4 | 5.2 | Middle |
| Cetin. et al, 2018 | 2000-2013 | Turkey | Western and Central Europe and North America | All site | 172 | 10 | 5.8 | Low |
| Slaught et al, 2019 | 2008-2015 | Botswana | East and Southern Africa | All site | 239 | 225 | 94.1 | Low |
| Castro et al, 2019 | 2009-2011 | Cameroon | West and Central Africa | All site | 943 | 680 | 72.1 | Low |
| Aliaga et al, 2019 | 2015-2018 | Peru | Latin America | Gastrointestinal KS | 13 | 12 | 92.3 | High |
| Kluger et al, 2019 | 2006-2018 | Finland | Western and Central Europe and North America | All site | 40 | 26 | 65.0 | Middle |
| Marcoval et al, 2019 | 1987-2016 | Spain | Western and Central Europe and North America | All site | 191 | 118 | 61.8 | Low |
| Huang et al, 2019 | 1996-2016 | China | Asia and the Pacific | All site | 55 | 17 | 30.9 | Middle |
| Baykal et al, 2019 | 2007-2017 | Turkey | Western and Central Europe and North America | All site | 137 | 11 | 8.0 | Low |
| Salman et al,, 2019 | 2006-2018 | Turkey | Western and Central Europe and North America | All site | 25 | 3 | 12.0 | High |
| Demba et al, 2020 | 2013-2016 | Kenya | East and Southern Africa | All site | 81 | 81 | 100.0 | Middle |
| Dhokotera et al, 2021* | 2004-2014 | South Africa | East and Southern Africa | All site | 791 | 786 | 99.4 | Low |
| Kalubul et al, 2021* | 2007-2014 | Zambia | East and Southern Africa | All site | 2891 | 1734 | 60.0 | Low |
| Oyucu et al, 2021* | 2008-2020 | Turkey | Western and Central Europe and North America | All site | 16 | 4 | 25.0 | High |
| Severin et al, 2021 | 2000-2017 | France | Western and Central Europe and North America | All site | 175 | 109 | 62.3 | Low |
| Güneş et al, 2022* | 2010-2020 | Turkey | Western and Central Europe and North America | All site | 27 | 1 | 3.7 | High |
| Higgins et al, 2022* | Not available | Afghanistan | Asia and the Pacific | All site | 7 | 1 | 14.3 | High |
| Lamoureux et al, 2022* | 1969-2019 | French Guiana | Latin America | All site | 52 | 30 | 57.7 | Middle |
| Malmström et al, 2022* | 1988-2017 | Sweden | Western and Central Europe and North America | All site | 292 | 184 | 63.0 | Low |
| Motlhale et al, 2022* | 1995-2016 | South Africa | East and Southern Africa | All site | 1275 | 1225 | 96.1 | Low |
| Sabourin, et al, 2022* | 2013-2016 | Zimbabwe | East and Southern Africa | All site | 520 | 520 | 100.0 | Low |
| Silverstein et al, 2022* | 2006-2015 | Malawi | East and Southern Africa | All site | 207 | 188 | 90.8 | Low |
| Sethomo et al, 2022* | 2001-2018 | Botswana | East and Southern Africa | All site | 115 | 5 | 4.4 | Low |
| Maldonado et al, 2023* | 2011-2020 | Colombia | Latin America | Gastrointestinal KS | 24 | 21 | 87.5 | High |
| AlOtaibi et al, 2023* | 2011-2022 | Saudi Arabia | Middle East and North Africa | All site | 6 | 6 | 100.0 | High |
| Campbell et al, 2023* | 2011-2020 | Malawi and Tanzania | East and Southern Africa | All site | 171 | 149 | 87.1 | Low |
| Caro-Vegas et al, 2023* | 2013-2019 | Malawi | East and Southern Africa | All site | 70 | 56 | 80.0 | Middle |
| Dinh et al, 2023* | 1983-2017 | Sweden | Western and Central Europe and North America | All site | 324 | 202 | 62.3 | Low |
| Desai et al, 2023* | 2004-2018 | USA | Western and Central Europe and North America | All site | 10027 | 6719 | 67.0 | Low |
| Guevara et al, 2023* | 2016-2020 | USA | Western and Central Europe and North America | lung KS | 460 | 130 | 28.3 | Low |
| Hagenimana et al, 2023* | 2007-2018 | Rwanda | East and Southern Africa | All site | 305 | 258 | 84.6 | Low |
| Hao et al, 2023* | 2015-2021 | USA | Western and Central Europe and North America | Foot and Ankle KS | 11 | 4 | 36.4 | High |
| Libson et al, 2023* | 2005-2020 | USA | Western and Central Europe and North America | All site | 91 | 72 | 79.1 | Middle |
| Lurain et al, 2023* | 2012-2014 | USA | Western and Central Europe and North America | All site | 19 | 12 | 63.2 | High |
| Ismaili et al, 2023* | 2010-2018 | Morocco | Middle East and North Africa | All site | 16 | 2 | 12.5 | High |
| Motlhale et al, 2023* | 1995-2016 | South Africa | East and Southern Africa | All site | 1237 | 1193 | 96.4 | Low |
| Pessoa et al, 2023* | 2017-2022 | Portugal | Western and Central Europe and North America | All site | 15 | 2 | 13.3 | High |
| Phipps et al, 2023* | 2012-2019 | Uganda | East and Southern Africa | All site | 200 | 166 | 83.0 | Low |
| Rasmussen et al, 2023* | 2000-2020 | France | Western and Central Europe and North America | All site | 33 | 29 | 87.9 | Middle |
| Ramaswami et al, 2023* | 2011-2022 | USA | Western and Central Europe and North America | All site | 48 | 47 | 97.9 | Middle |
| Sengayi et al, 2023* | 1995-2016 | South Africa | East and Southern Africa | All site | 1268 | 1215 | 95.8 | Low |
| Valcarcel et al, 2023* | 2000-2014 | Peru | Latin America | All site | 176 | 95 | 54.0 | Low |
| Yang er al 2023* | 2017-2022 | China | Asia and the Pacific | All site | 32 | 10 | 31.3 | Middle |
| Zhou et al, 2023* | 2012 | USA | Western and Central Europe and North America | All site | 296 | 73 | 24.7 | Low |
| Russo et al, 2024* | 1993-2022 | Italy | Western and Central Europe and North America | All site | 86 | 30 | 34.9 | Middle |
| Manase et al, 2024* | 2010-2023 | Malawi | East and Southern Africa | All site | 52 | 42 | 80.8 | Middle |
| Morais et al, 2024* | Not available | Brazil | Latin America | All site | 106 | 40 | 37.7 | Low |
| Saberian et al, 2024* | 2016-2021 | USA | Western and Central Europe and North America | All site | 32 | 30 | 93.8 | Middle |
| Swan et al, 2024* | 2007-2010 | Uganda | East and Southern Africa | All site | 65 | 56 | 86.2 | Middle |

* Newly added studies based on the previously published literature review.

 Figure. S1. Funnel plots for evaluating publication bias in the meta-analyses of the association between HIV infection and the risk of (A) cervical cancer, (B) non-Hodgkin lymphoma, and (C) Kaposi sarcoma

Table S4. Subgroup analysis for pooled risk ratio by UNAIDS regions

| UNAIDS region | Cervical cancer | | | | | Non-Hodgkin lymphoma | | | | |
| --- | --- | --- | --- | --- | --- | --- | --- | --- | --- | --- |
|  | Number of estimates | Pooled RR (95%CI) | Model | *I*^2^ value (%) | *p*-value | Number of estimates | Pooled RR (95%CI) | Model | *I*^2^ value (%) | *p*-value |
| Global | 37 | 4.85 (3.69, 6.37) | Random | 98.2 | < 0.001 | 55 | 21.14 (15.86, 28.19) | Random | 99.7 | < 0.001 |
| Asia and the Pacific | 6* | 9.83 (4.24, 22.80) | Random | 91.6 | < 0.001 | 14* | 25.83 (17.18, 38.84) | Random | 98.5 | < 0.001 |
| Caribbean | 1 | 4.85 (3.69, 6.37) | Random | 98.2 | < 0.001 | 0 | 21.14 (15.86, 28.19) | Random | 99.7 | < 0.001 |
| Eastern Europe and Central Asia | 0 | 4.85 (3.69, 6.37) | Random | 98.2 | < 0.001 | 0 | 21.14 (15.86, 28.19) | Random | 99.7 | < 0.001 |
| East and Southern Africa | 7* | 2.86 (2.26, 3.62) | Random | 67.7 | < 0.001 | 5* | 4.56 (2.20, 9.45) | Random | 97.5 | < 0.001 |
| Latin America | 0 | 4.85 (3.69, 6.37) | Random | 98.2 | < 0.001 | 2 | 21.14 (15.86, 28.19) | Random | 99.7 | < 0.001 |
| Middle East and North Africa | 0 | 4.85 (3.69, 6.37) | Random | 98.2 | < 0.001 | 0 | 21.14 (15.86, 28.19) | Random | 99.7 | < 0.001 |
| West and Central Africa | 6* | 3.23 (1.82, 5.74) | Random | 83.1 | < 0.001 | 2 | 21.14 (15.86, 28.19) | Random | 99.7 | < 0.001 |
| Western and Central Europe and North America | 17* | 4.77 (3.52, 6.46) | Random | 92.0 | < 0.001 | 32* | 27.67 (19.27, 39.74) | Random | 99.8 | < 0.001 |
| * We calculated a pooled RR value for these UNAIDS regions in specific period when the number of estimates reaches five. Otherwise, region pooled RR is equal to the corresponding global one | | | | | | | | | | |

Abbreviations: UNAIDS, the Joint United Nations Programme on HIV and AIDS; RR, risk ratio; CI, confidence interval

Table S5. Subgroup analysis for pooled risk ratio by UNAIDS regions (excluding studies identified as having higher potential for publication bias)

| UNAIDS region | Cervical cancer | | | | Non-Hodgkin lymphoma | | | | Kaposi sarcoma | | | |
| --- | --- | --- | --- | --- | --- | --- | --- | --- | --- | --- | --- | --- |
|  | Number of estimates | Pooled RR (95%CI) | *I*^2^ value (%) | *p*-value | Number of estimates | Pooled RR (95%CI) | *I*^2^ value (%) | *p*-value | Number of estimates | Pooled PAF (95%CI) | *I*^2^ value (%) | *p*-value |
| Global | 17 | 3.91 (2.97, 5.17) | 99.0 | < 0.001 | 54 | 20.63 (15.45, 27.55) | 99.7 | < 0.001 | 68 | 60.7 (50.8, 70.1) | 99.5 | < 0.001 |
| Asia and the Pacific | 4 | 3.91 (2.97, 5.17) | 99.0 | < 0.001 | 14* | 25.83 (17.18, 38.84) | 98.5 | < 0.001 | 5* | 35.4 (17.5, 55.7) | 90.8 | < 0.001 |
| Caribbean | 1 | 3.91 (2.97, 5.17) | 99.0 | < 0.001 | 0 | 20.63 (15.45, 27.55) | 99.7 | < 0.001 | 0 | 60.7 (50.8, 70.1) | 99.5 | < 0.001 |
| Eastern Europe and Central Asia | 0 | 3.91 (2.97, 5.17) | 99.0 | < 0.001 | 0 | 20.63 (15.45, 27.55) | 99.7 | < 0.001 | 0 | 60.7 (50.8, 70.1) | 99.5 | < 0.001 |
| East and Southern Africa | 3 | 3.91 (2.97, 5.17) | 99.0 | < 0.001 | 5* | 4.56 (2.20, 9.45) | 97.5 | < 0.001 | 27* | 86.8 (79.1, 92.9) | 99.2 | < 0.001 |
| Latin America | 0 | 3.91 (2.97, 5.17) | 99.0 | < 0.001 | 2 | 20.63 (15.45, 27.55) | 99.7 | < 0.001 | 4 | 60.7 (50.8, 70.1) | 99.5 | < 0.001 |
| Middle East and North Africa | 0 | 3.91 (2.97, 5.17) | 99.0 | < 0.001 | 0 | 20.63 (15.45, 27.55) | 99.7 | < 0.001 | 5* | 3.3 (1.4, 5.8) | 0 | 0.632 |
| West and Central Africa | 1 | 3.91 (2.97, 5.17) | 99.0 | < 0.001 | 2 | 20.63 (15.45, 27.55) | 99.7 | < 0.001 | 3 | 60.7 (50.8, 70.1) | 99.5 | < 0.001 |
| Western and Central Europe and North America | 9* | 3.80 (3.02, 4.78) | 93.4 | < 0.001 | 31* | 26.77 (18.53, 38.68) | 99.8 | < 0.001 | 24* | 44.8 (30.0, 60.1) | 99.6 | < 0.001 |
| * We calculated a pooled RR value for these UNAIDS regions in specific period when the number of estimates reaches five. Otherwise, region pooled RR is equal to the corresponding global one  Abbreviations: UNAIDS, the Joint United Nations Programme on HIV and AIDS; RR, risk ratio; CI, confidence interval | | | | | | | | | | | | |

Table S6. Estimated population attributable fraction of HIV for AIDS-defining cancers in 2022, by country

| UNAIDS region | Country | Cervical cancer | | | Non-Hodgkin lymphoma | | |
| --- | --- | --- | --- | --- | --- | --- | --- |
|  |  | Number of new cases (95% CI) | Population attributable fraction of HIV (95% CI) | Number of cases attributable to HIV (95% CI) | Number of new cases (95% CI) | Population attributable fraction of HIV (95% CI) | Number of cases attributable to HIV (95% CI) |
| Asia and the Pacific | Papua New Guinea | 1057(652-1463) | 10.1 (3.9-21.7) | 105(37-246) | 510(0-3960) | 19.8(13.5-27.6) | 98(0-824) |
|  | Thailand | 8667(8286-9042) | 6.6 (2.5-15.1) | 572(218-1296) | 6602(5962-7233) | 19.2(13.2-27.2) | 1268(864-1811) |
|  | Cambodia | 1275(1137-1415) | 5.1 (1.9-11.9) | 65(24-153) | 639(531-749) | 13.7(9.2-19.9) | 87(56-131) |
|  | Myanmar | 7029(6665-7398) | 4.6 (1.7-10.8) | 323(119-765) | 1960(1768-2153) | 14.2(9.3-20.9) | 278(179-412) |
|  | Fiji | 165(59-270) | 3.1 (1-8.3) | 5(1-15) | 52(0-423) | 9(4.5-15.6) | 4(0-43) |
|  | Viet Nam | 4612(4343-4881) | 1.7 (0.6-4.1) | 78(29-187) | 3517(3053-3978) | 7.4(4.9-11) | 260(168-393) |
|  | Lao People's Democratic Republic | 401(369-432) | 2.2 (0.8-5.3) | 9(3-21) | 267(224-308) | 7.9(5.2-11.8) | 21(14-32) |
|  | Malaysia | 1916(1375-2464) | 1.1 (0.4-2.6) | 20(7-52) | 2156(154-4183) | 7.4(4.9-11) | 158(10-357) |
|  | Australia | 897(776-1020) | 0.3 (0.1-0.8) | 3(1-7) | 5897(4908-6907) | 3.3(2.2-5) | 194(122-304) |
|  | Indonesia | 37280(10191-64412) | 1.5 (0.6-3.7) | 546(112-1649) | 16344(1831-30881) | 6.2(4.1-9.3) | 997(109-2155) |
|  | Pakistan | 4762(4465-5063) | 0.6 (0.2-1.4) | 27(10-66) | 7295(6564-8037) | 4(2.6-6) | 291(189-444) |
|  | Singapore | 353(284-421) | 0.2 (0.1-0.6) | 1(0-2) | 1150(902-1393) | 3.5(2.2-5.3) | 40(24-64) |
|  | Timor-Leste | 77(53-101) | 1.2 (0.5-3) | 1(0-2) | 38(22-54) | 4.3(2.8-6.5) | 2(1-3) |
|  | Bhutan | 49(49-49) | 1.6 (0.6-4) | 1(0-2) | 9(9-9) | 4.6(2.9-7.2) | 0(0-1) |
|  | New Zealand | 149(104-195) | 0.2 (0.1-0.6) | 0(0-1) | 950(732-1177) | 2.1(1.3-3.2) | 20(12-32) |
|  | Philippines | 8545(8154-8948) | 0.2 (0.1-0.6) | 21(8-53) | 4982(4341-5644) | 4.7(3-7.2) | 234(148-363) |
|  | Nepal | 2169(2152-2185) | 1 (0.4-2.3) | 21(8-51) | 565(543-587) | 3.2(2.1-4.8) | 18(12-27) |
|  | Afghanistan | 1214(431-1995) | 0.1 (0-0.4) | 1(0-8) | 592(0-5123) | 0.3(0-6.7) | 0(0-166) |
|  | Sri Lanka | 1580(1424-1733) | 0.1 (0-0.3) | 2(1-4) | 1024(774-1269) | 0.7(0.4-1) | 7(4-11) |
|  | Mongolia | 361(285-436) | 0.1 (0-0.2) | 0(0-1) | 45(0-107) | 0.7(0.4-1.1) | 0(0-1) |
|  | Bangladesh | 9640(9561-9718) | 0.1 (0-0.2) | 6(2-16) | 3095(2960-3231) | 0.3(0.2-0.4) | 9(6-13) |
| Caribbean | Haiti | 872(451-1285) | 6.9 (4.8-9.6) | 59(28-100) | 458(0-1828) | 25.1(19.3-31.9) | 115(0-476) |
|  | Bahamas | 42(0-100) | 4 (2.8-5.5) | 2(0-5) | 29(0-249) | 19.6(14.8-25.1) | 6(0-62) |
|  | Suriname | 82(0-212) | 5.4 (3.7-7.5) | 4(0-12) | 39(0-310) | 23.8(18.3-30.2) | 9(0-77) |
|  | Barbados | 36(0-89) | 2.6 (1.8-3.6) | 1(0-2) | 34(0-295) | 15.1(11.2-20.1) | 5(0-47) |
|  | Guyana | 129(0-285) | 6.3 (3.8-9.7) | 8(0-21) | 48(0-380) | 26.3(18.1-35.6) | 12(0-104) |
|  | Jamaica | 376(229-526) | 3.9 (2.7-5.4) | 14(8-23) | 239(0-513) | 18.1(13.8-23.2) | 43(0-98) |
|  | Dominican Republic | 970(699-1241) | 3.5 (2.4-5) | 34(21-52) | 304(0-1057) | 16.4(12-21.7) | 49(0-178) |
|  | Belize | 33(0-95) | 4.1 (2.8-5.7) | 1(0-4) | 7(0-66) | 19.4(14.9-24.7) | 1(0-13) |
|  | Cuba | 1122(798-1442) | 0.6 (0.4-0.9) | 7(4-11) | 1154(0-2400) | 8.2(6-11.1) | 94(0-210) |
|  | Saint Lucia | 20(0-76) | 1.6 (1.1-2.3) | 0(0-1) | 17(0-138) | 8.5(6.3-11.3) | 1(0-12) |
| Eastern Europe and Central Asia | Republic of Moldova | 420(319-523) | 1.8 (1.2-2.6) | 8(5-12) | 310(233-388) | 12(8.6-16.1) | 37(24-54) |
|  | Belarus | 670(525-815) | 0.9 (0.6-1.3) | 6(4-9) | 1024(909-1140) | 6.6(4.6-9.3) | 67(46-97) |
|  | Azerbaijan | 546(334-758) | 0.3 (0.2-0.4) | 1(1-2) | 420(0-1047) | 2.5(1.7-3.4) | 10(0-28) |
|  | Georgia | 331(187-478) | 0.6 (0.4-0.8) | 2(1-3) | 300(0-884) | 5.6(4.1-7.4) | 17(0-52) |
|  | Tajikistan | 278(118-432) | 0.5 (0.4-0.8) | 1(1-3) | 193(0-1528) | 4.1(3-5.6) | 8(0-65) |
|  | Kyrgyzstan | 480(258-699) | 0.7 (0.5-1) | 4(2-6) | 99(0-377) | 4.6(3.4-6.2) | 5(0-18) |
|  | Armenia | 155(28-284) | 0.6 (0.4-0.8) | 1(0-2) | 113(0-928) | 5.1(3.8-6.9) | 6(0-49) |
|  | Kazakhstan | 1824(1662-1980) | 0.7 (0.5-1) | 12(8-18) | 476(315-631) | 5.3(3.8-7.2) | 25(15-38) |
|  | Uzbekistan | 2652(2091-3235) | 0.8 (0.5-1.1) | 20(13-30) | 603(77-1149) | 4.3(3.2-5.7) | 25(3-52) |
|  | Albania | 171(139-202) | 0.1 (0.1-0.2) | 0(0-0) | 99(68-129) | 1.2(0.9-1.7) | 1(1-2) |
|  | Montenegro | 58(35-82) | 0.1 (0-0.1) | 0(0-0) | 57(28-86) | 1.6(1.1-2.3) | 1(0-2) |
|  | North Macedonia | 107(69-144) | 0 (0-0) | 0(0-0) | 82(47-116) | 0.7(0.5-1) | 1(0-1) |
|  | Bosnia and Herzegovina | 263(206-320) | 0 (0-0) | 0(0-0) | 213(156-269) | 0.4(0.3-0.7) | 1(1-1) |
| East and Southern Africa | Namibia | 350(278-420) | 21.7 (15.6-28.3) | 75(51-105) | 151(35-264) | 34(14.7-55.4) | 49(9-111) |
|  | Botswana | 453(366-542) | 31 (23.2-38.9) | 140(98-188) | 98(2-196) | 41.6(19.3-62.8) | 39(1-93) |
|  | Eswatini | 416(335-499) | 38.2 (29.1-46.9) | 158(113-210) | 28(0-110) | 50.3(25.2-70.7) | 13(0-59) |
|  | Mozambique | 5457(5126-5791) | 21.7 (15.7-28.2) | 1182(852-1552) | 1368(1027-1713) | 34.4(14.9-55.6) | 465(196-809) |
|  | Zambia | 3641(3385-3896) | 20.9 (15.1-27.4) | 762(546-1001) | 408(236-578) | 33.2(14.2-54.4) | 132(50-252) |
|  | Lesotho | 598(480-712) | 32.2 (24-40.6) | 191(134-257) | 50(0-212) | 43.3(20.3-64.5) | 20(0-100) |
|  | South Africa | 10540(9954-11116) | 28.2 (20.9-35.8) | 2970(2192-3787) | 3263(2925-3597) | 37.2(16.5-58.4) | 1213(538-1930) |
|  | Uganda | 6939(6579-7289) | 11.9 (8.3-16.2) | 826(575-1126) | 2270(1885-2645) | 17.3(6.5-33.2) | 391(146-768) |
|  | Zimbabwe | 3520(3277-3763) | 21.1 (15.2-27.8) | 745(531-986) | 1070(788-1352) | 30.3(12.6-51) | 322(131-579) |
|  | Malawi | 4703(4379-5031) | 14.8 (10.4-20.2) | 697(483-957) | 1308(993-1627) | 22.3(8.8-40.7) | 289(110-559) |
|  | United Republic of Tanzania | 10873(10375-11372) | 9.9 (6.9-13.5) | 1079(748-1478) | 1344(880-1809) | 14.1(5.2-28.1) | 187(64-405) |
|  | Angola | 2823(2750-2895) | 3.5 (2.3-5) | 98(64-145) | 792(757-826) | 5(1.7-11.4) | 40(13-94) |
|  | Kenya | 5847(5520-6181) | 8.5 (5.8-11.8) | 500(340-697) | 1682(1359-2012) | 12.1(4.4-24.7) | 203(72-426) |
|  | Rwanda | 866(756-980) | 6.1 (4.1-8.5) | 53(35-75) | 316(177-461) | 9.1(3.2-19.4) | 28(9-68) |
|  | South Sudan | 749(498-1003) | 3.5 (2.3-5.1) | 26(15-42) | 275(0-2250) | 5.3(1.8-11.9) | 12(0-155) |
|  | Ethiopia | 8172(7802-8543) | 2.1 (1.4-3.1) | 174(114-257) | 3574(3088-4060) | 3.1(1.1-7.2) | 110(37-261) |
|  | Eritrea | 196(144-248) | 1.3 (0.8-1.9) | 3(2-4) | 131(95-166) | 2.1(0.7-4.9) | 3(1-7) |
|  | Mauritius | 136(94-177) | 1.6 (1-2.3) | 2(1-3) | 47(0-109) | 3.9(1.3-8.9) | 2(0-6) |
|  | Madagascar | 4061(3827-4292) | 0.7 (0.4-1) | 27(16-41) | 908(375-1432) | 1.3(0.4-3.2) | 12(3-33) |
|  | Comoros | 163(151-175) | 0.1 (0-0.1) | 0(0-0) | 31(11-51) | 0.1(0-0.3) | 0(0-0) |
| Latin America | Panama | 372(123-625) | 1.7 (1.2-2.4) | 6(2-12) | 234(0-726) | 15.2(11.5-19.7) | 35(0-117) |
|  | Colombia | 4571(3728-5402) | 0.7 (0.5-1.1) | 34(21-53) | 3866(2235-5474) | 10.7(7.4-14.8) | 407(217-668) |
|  | Uruguay | 376(305-450) | 1.5 (0.9-2.3) | 5(3-9) | 485(327-648) | 10.7(7.2-15.1) | 52(30-83) |
|  | Chile | 1556(1075-2038) | 0.6 (0.4-0.9) | 10(6-15) | 1561(324-2800) | 9.8(7.3-13) | 150(30-299) |
|  | Costa Rica | 341(313-370) | 0.5 (0.3-0.7) | 2(1-2) | 413(261-565) | 8.2(6-11) | 33(19-52) |
|  | Peru | 4812(3847-5746) | 0.7 (0.5-1) | 34(22-51) | 3904(1760-5979) | 7.9(5.7-10.6) | 304(131-523) |
|  | Paraguay | 1114(746-1496) | 1.1 (0.7-1.6) | 12(7-19) | 409(0-1362) | 8.1(5.7-11) | 32(0-116) |
|  | Argentina | 4692(3976-5407) | 1 (0.7-1.4) | 48(29-78) | 3824(1379-6265) | 7.1(5.3-9.4) | 271(73-589) |
|  | Venezuela | 3972(3271-4675) | 0.9 (0.6-1.3) | 35(23-52) | 2060(191-3933) | 8(5.9-10.7) | 163(15-340) |
|  | Mexico | 10353(8845-11878) | 0.5 (0.4-0.7) | 54(36-79) | 9014(4343-13739) | 7(5.2-9.4) | 625(287-1064) |
|  | Ecuador | 1794(1382-2216) | 0.9 (0.6-1.3) | 16(10-25) | 1469(461-2499) | 6.7(5-9.1) | 98(30-183) |
|  | El Salvador | 627(367-890) | 1.2 (0.8-1.6) | 7(4-12) | 183(0-619) | 8.5(6.4-11.3) | 15(0-55) |
|  | Nicaragua | 720(373-1064) | 0.5 (0.3-0.8) | 4(2-7) | 294(0-881) | 4.3(3-6.3) | 13(0-42) |
|  | Honduras | 917(776-1055) | 0.8 (0.6-1.1) | 7(5-11) | 515(322-705) | 4.9(3.6-6.6) | 25(15-39) |
|  | Guatemala | 1762(1267-2237) | 0.8 (0.5-1.1) | 13(8-20) | 362(0-901) | 5.1(3.8-6.8) | 18(0-49) |
|  | Bolivia | 2208(1619-2808) | 0.7 (0.5-1.1) | 16(10-25) | 286(0-1248) | 6.7(4.9-9.1) | 19(0-87) |
| Middle East and North Africa | Djibouti | 71(44-97) | 3.4 (2-5.5) | 2(1-5) | 45(31-59) | 15.3(9.3-23) | 7(3-13) |
|  | Egypt | 1301(1144-1458) | 1.2 (0.8-1.8) | 16(11-24) | 8855(7868-9842) | 8.5(6.2-11.4) | 747(538-1041) |
|  | Sudan | 1234(1060-1410) | 0.6 (0.3-0.9) | 7(4-11) | 1528(1139-1924) | 3.1(1.9-4.8) | 47(27-77) |
|  | Libya | 278(190-368) | 0.4 (0.3-0.6) | 1(1-2) | 334(146-523) | 2.5(1.9-3.5) | 8(4-15) |
|  | Iran (Islamic Republic of) | 1265(1123-1405) | 0.2 (0.1-0.3) | 3(1-4) | 3656(3169-4136) | 1.6(0.8-2.4) | 60(29-89) |
|  | Oman | 90(55-126) | 0.2 (0.2-0.3) | 0(0-0) | 266(134-399) | 1.6(1.2-2.2) | 4(2-7) |
|  | Tunisia | 414(308-518) | 0.2 (0.1-0.3) | 1(0-1) | 864(562-1158) | 1.6(1.1-2.3) | 13(8-22) |
|  | Lebanon | 144(93-195) | 0.1 (0-0.1) | 0(0-0) | 645(446-841) | 1.4(1-2) | 9(5-14) |
|  | Yemen | 213(159-265) | 0.3 (0.1-0.4) | 1(0-1) | 854(640-1064) | 1.3(0.8-1.9) | 11(7-17) |
|  | Morocco | 2643(2412-2872) | 0.3 (0.2-0.4) | 7(5-10) | 2380(2005-2751) | 1.6(1.2-2.2) | 38(26-54) |
|  | Algeria | 1797(1631-1967) | 0.3 (0.2-0.4) | 5(3-7) | 2223(1845-2608) | 1.5(1.1-2) | 32(23-46) |
|  | Qatar | 25(0-50) | 0.1 (0.1-0.1) | 0(0-0) | 96(0-224) | 0.7(0.5-0.9) | 1(0-2) |
|  | Saudi Arabia | 332(259-407) | 0.1 (0-0.1) | 0(0-0) | 1908(1552-2277) | 0.7(0.5-1) | 14(10-20) |
|  | Somalia | 1166(1106-1228) | 0.4 (0.2-0.5) | 4(3-6) | 464(374-557) | 1.6(1.1-2.2) | 7(5-11) |
|  | Kuwait | 61(31-90) | 0 (0-0.1) | 0(0-0) | 258(135-377) | 0.6(0.4-0.8) | 1(1-3) |
|  | Iraq | 312(242-382) | 0 (0-0) | 0(0-0) | 1934(1601-2268) | 0.2(0.1-0.3) | 4(3-6) |
|  | Jordan | 133(87-181) | 0 (0-0) | 0(0-0) | 543(368-722) | 0.2(0.1-0.2) | 1(1-1) |
|  | Syrian Arab Republic | 207(66-349) | 0 (0-0) | 0(0-0) | 920(350-1491) | 0.1(0.1-0.1) | 1(0-1) |
| West and Central Africa | Gabon | 271(200-341) | 9.8 (3.7-19.2) | 26(10-54) | 78(0-157) | 40(31.6-48.6) | 31(0-65) |
|  | Togo | 512(399-627) | 5.1 (1.9-10.5) | 26(9-55) | 249(0-988) | 27.4(20.8-34.8) | 67(0-279) |
|  | Burundi | 1455(1062-1847) | 2.9 (1.1-6) | 42(11-110) | 377(121-632) | 17.8(13.4-23.2) | 67(17-145) |
|  | Côte d'Ivoire | 2360(2156-2562) | 6.6 (2.5-13.1) | 155(58-310) | 958(705-1209) | 31.7(25-39.1) | 302(208-415) |
|  | Congo | 397(320-477) | 9 (3.4-18) | 35(13-74) | 50(0-119) | 39.7(30.2-49.4) | 20(0-49) |
|  | Nigeria | 13677(13196-14145) | 4.1 (1.5-8.4) | 564(209-1141) | 5195(4407-5964) | 23.1(17.9-29.2) | 1197(889-1571) |
|  | Guinea-Bissau | 225(21-422) | 6.9 (2.6-13.8) | 15(1-40) | 42(0-112) | 34.2(27.1-42.3) | 14(0-39) |
|  | Ghana | 3073(2844-3304) | 4.3 (1.6-8.8) | 131(49-272) | 1375(1047-1706) | 24(18.2-30.7) | 328(225-457) |
|  | Cabo Verde | 46(32-60) | 2.1 (0.8-4.4) | 1(0-2) | 12(0-29) | 17.1(12.8-22.5) | 2(0-5) |
|  | Burkina Faso | 988(974-1002) | 1.9 (0.7-4) | 19(7-40) | 380(368-392) | 12.1(9-16.1) | 46(34-61) |
|  | Chad | 1111(1034-1188) | 3 (1.1-6.3) | 33(12-70) | 349(223-474) | 18.2(13.5-23.8) | 63(37-96) |
|  | Gambia | 325(246-406) | 4.2 (1.6-8.9) | 14(5-30) | 38(0-121) | 24.6(18-32.1) | 9(0-31) |
|  | Liberia | 717(605-832) | 2.7 (1-5.8) | 20(7-42) | 148(0-315) | 16.3(11.6-22.1) | 24(0-55) |
|  | Mali | 2436(2207-2668) | 2.5 (0.9-5.4) | 62(23-133) | 410(221-602) | 15.4(10.9-21.1) | 63(31-105) |
|  | Guinea | 2550(2263-2835) | 3.8 (1.4-7.8) | 96(35-200) | 276(95-456) | 22.2(16.9-28.5) | 61(20-109) |
|  | Benin | 700(582-820) | 2.2 (0.8-4.8) | 15(6-34) | 174(38-312) | 13.7(9.3-18.6) | 23(5-47) |
|  | Sierra Leone | 487(289-684) | 3.6 (1.3-7.6) | 17(6-40) | 67(36-98) | 21.3(16-27.7) | 14(7-23) |
|  | Democratic Republic of the Congo | 8755(4495-13022) | 1.9 (0.7-4.2) | 165(51-407) | 2106(0-16417) | 13(9.2-17.5) | 265(0-2202) |
|  | Senegal | 2091(7-4143) | 1 (0.4-2.1) | 19(0-58) | 331(0-808) | 7.5(5.5-10.1) | 25(0-64) |
|  | Niger | 625(515-736) | 0.5 (0.2-1.1) | 3(1-7) | 363(178-548) | 4(3-5.4) | 14(7-25) |
|  | Mauritania | 464(90-850) | 0.5 (0.2-1.1) | 2(0-6) | 97(0-268) | 4.5(3.2-6.2) | 4(0-13) |
| Western and Central Europe and North America | Estonia | 121(91-152) | 1.8 (1.2-2.6) | 2(1-3) | 256(208-303) | 14.9(10.6-20.5) | 38(25-54) |
|  | Portugal | 897(718-1072) | 1.1 (0.7-1.6) | 10(6-15) | 2438(2134-2734) | 12.2(8.5-17.1) | 296(204-422) |
|  | United States | 13923(13493-14371) | 0.7 (0.4-1) | 95(61-143) | 77962(77043-78921) | 10.5(7.2-14.8) | 8154(5626-11560) |
|  | Spain | 2020(1738-2298) | 0.5 (0.3-0.7) | 9(6-14) | 9071(8392-9741) | 8.8(6-12.6) | 798(543-1143) |
|  | France | 3187(2840-3541) | 0.9 (0.6-1.3) | 29(19-43) | 16978(15978-18000) | 8.9(6.1-12.7) | 1505(1028-2158) |
|  | Italy | 2481(2065-2903) | 0.5 (0.4-0.8) | 14(9-21) | 15582(14620-16555) | 6.8(4.6-9.8) | 1053(720-1539) |
|  | Latvia | 238(188-289) | 1 (0.7-1.5) | 2(2-4) | 253(152-355) | 10.5(7.4-14.7) | 26(15-43) |
|  | Switzerland | 268(166-372) | 0.5 (0.3-0.7) | 1(1-2) | 1901(1589-2220) | 5.8(4-8.4) | 110(73-165) |
|  | Luxembourg | 22(0-45) | 0.6 (0.4-0.9) | 0(0-0) | 93(44-143) | 6.8(4.7-9.7) | 6(3-11) |
|  | Malta | 13(5-21) | 0.3 (0.2-0.4) | 0(0-0) | 131(102-160) | 4.7(3.2-7) | 6(4-10) |
|  | Israel | 330(267-392) | 0.3 (0.2-0.4) | 1(1-2) | 1679(1361-1994) | 3.6(2.4-5.4) | 61(39-94) |
|  | Ireland | 242(192-291) | 0.5 (0.3-0.7) | 1(1-2) | 1029(917-1139) | 4.9(3.3-7.1) | 51(34-74) |
|  | Greece | 473(345-596) | 0.2 (0.1-0.3) | 1(1-2) | 1663(1481-1838) | 4.1(2.7-6) | 68(45-101) |
|  | Netherlands | 757(670-844) | 0.2 (0.2-0.3) | 2(1-3) | 4384(4133-4634) | 4.2(2.8-6) | 183(124-266) |
|  | Lithuania | 296(231-359) | 0.3 (0.2-0.4) | 1(0-1) | 306(237-373) | 4(2.7-5.9) | 12(8-19) |
|  | Iceland | 19(10-29) | 0.3 (0.2-0.4) | 0(0-0) | 66(48-85) | 3(2-4.4) | 2(1-3) |
|  | Romania | 3373(2995-3745) | 0.3 (0.2-0.4) | 10(6-14) | 1757(1508-2003) | 2.8(1.9-4.1) | 49(32-73) |
|  | Czechia | 658(576-739) | 0 (0-0.1) | 0(0-0) | 1764(1636-1893) | 1.2(0.8-1.8) | 21(14-32) |
|  | Serbia | 907(763-1050) | 0.1 (0-0.1) | 1(0-1) | 957(753-1160) | 1.6(1.1-2.4) | 15(10-24) |
|  | Slovenia | 119(91-148) | 0 (0-0.1) | 0(0-0) | 478(415-544) | 1.3(0.9-2) | 6(4-10) |
|  | Bulgaria | 876(761-991) | 0.1 (0.1-0.1) | 1(0-1) | 492(430-553) | 1.6(1.1-2.4) | 8(5-13) |
|  | Slovakia | 557(415-699) | 0 (0-0.1) | 0(0-0) | 683(589-777) | 0.9(0.6-1.3) | 6(4-9) |

Table S6. Estimated population attributable fraction of HIV for AIDS-defining cancers in 2022, by country (continued)

| UNAIDS region | Country | Kaposi sarcoma | | | AIDS-defining cancers | | |
| --- | --- | --- | --- | --- | --- | --- | --- |
|  |  | Number of new cases (95% CI) | Population attributable fraction of HIV (95% CI) | Number of cases attributable to HIV (95% CI) | Number of new cases (95% CI) | Number of cases attributable to HIV (95% CI) | Population attributable fraction of HIV (95% CI) |
| Asia and the Pacific | Papua New Guinea | 8(6-10) | 26.3(12.3-44.2) | 2(1-4) | 1576(661-5431) | 210(42-1026) | 13.5(5.1-24.1) |
|  | Thailand | 70(0-196) | 26.3(12.3-44.2) | 17(0-61) | 15343(14324-16353) | 1865(1115-3094) | 12.1(7.3-20.2) |
|  | Cambodia | 1(0-1) | 26.3(12.3-44.2) | 0(0-1) | 1914(1670-2165) | 153(82-282) | 8(4.4-14.4) |
|  | Myanmar | 15(0-119) | 26.3(12.3-44.2) | 3(0-35) | 9018(8464-9586) | 608(313-1173) | 6.7(3.5-13) |
|  | Fiji | 0(0-0) | 26.3(12.3-44.2) | 0(0-0) | 217(59-693) | 10(1-55) | 4.6(1.3-10.4) |
|  | Viet Nam | 165(0-338) | 26.3(12.3-44.2) | 41(0-109) | 8295(7549-9038) | 383(234-627) | 4.6(2.9-7.4) |
|  | Lao People's Democratic Republic | 2(1-3) | 26.3(12.3-44.2) | 1(0-1) | 670(595-742) | 30(18-53) | 4.5(2.6-7.9) |
|  | Malaysia | 26(0-51) | 26.3(12.3-44.2) | 7(0-17) | 4100(1557-6680) | 186(30-406) | 4.4(2-7.3) |
|  | Australia | 48(0-190) | 26.3(12.3-44.2) | 12(0-57) | 6841(5683-8118) | 213(128-342) | 3.1(2-4.7) |
|  | Indonesia | 116(0-236) | 26.3(12.3-44.2) | 29(0-77) | 53753(12050-95456) | 1579(282-3795) | 3(1.7-5.6) |
|  | Pakistan | 79(0-502) | 26.3(12.3-44.2) | 20(0-146) | 12183(11132-13272) | 352(216-566) | 2.9(1.8-4.6) |
|  | Singapore | 5(0-20) | 26.3(12.3-44.2) | 1(0-6) | 1510(1192-1822) | 42(26-68) | 2.8(1.8-4.3) |
|  | Timor-Leste | 0(0-0) | 26.3(12.3-44.2) | 0(0-0) | 115(75-155) | 3(1-5) | 2.2(1.2-4.2) |
|  | Bhutan | 0(0-0) | 26.3(12.3-44.2) | 0(0-0) | 58(58-58) | 1(1-3) | 2.1(1-4.4) |
|  | New Zealand | 5(0-24) | 26.3(12.3-44.2) | 1(0-7) | 1106(842-1377) | 22(13-36) | 2(1.3-3.1) |
|  | Philippines | 13(0-80) | 26.3(12.3-44.2) | 3(0-23) | 13548(12514-14613) | 260(161-419) | 1.9(1.2-3.1) |
|  | Nepal | 0(0-0) | 26.3(12.3-44.2) | 0(0-0) | 2734(2695-2772) | 39(20-78) | 1.4(0.7-2.8) |
|  | Afghanistan | 25(0-215) | 26.3(12.3-44.2) | 6(0-64) | 1831(431-7333) | 11(0-217) | 0.5(0-4.2) |
|  | Sri Lanka | 0(0-0) | 26.3(12.3-44.2) | 0(0-0) | 2604(2198-3002) | 9(5-15) | 0.3(0.2-0.6) |
|  | Mongolia | 0(0-0) | 26.3(12.3-44.2) | 0(0-0) | 405(285-543) | 1(0-1) | 0.1(0.1-0.3) |
|  | Bangladesh | 0(0-0) | 26.3(12.3-44.2) | 0(0-0) | 12735(12521-12949) | 15(8-29) | 0.1(0.1-0.2) |
| Caribbean | Haiti | 59(0-457) | 57.8(49.8-65.7) | 34(0-263) | 1439(510-3243) | 242(34-664) | 16.3(6.1-26.5) |
|  | Bahamas | 5(0-42) | 57.8(49.8-65.7) | 3(0-24) | 75(0-390) | 10(0-91) | 14.8(3.1-23.6) |
|  | Suriname | 2(0-17) | 57.8(49.8-65.7) | 1(0-10) | 125(0-528) | 16(0-91) | 14.4(4.6-59.4) |
|  | Barbados | 6(0-50) | 57.8(49.8-65.7) | 3(0-29) | 82(0-399) | 16(0-60) | 14(2.2-59.4) |
|  | Guyana | 0(0-0) | 57.8(49.8-65.7) | 0(0-0) | 177(0-665) | 20(0-124) | 11.7(4.4-20.9) |
|  | Jamaica | 0(0-0) | 57.8(49.8-65.7) | 0(0-0) | 615(229-1039) | 57(9-120) | 9.1(3.9-12.8) |
|  | Dominican Republic | 33(0-274) | 57.8(49.8-65.7) | 19(0-158) | 1340(731-2366) | 121(24-305) | 8.7(3.1-16.3) |
|  | Belize | 0(0-0) | 57.8(49.8-65.7) | 0(0-0) | 41(0-161) | 3(0-17) | 7.5(3.1-12) |
|  | Cuba | 23(0-196) | 57.8(49.8-65.7) | 13(0-114) | 2326(832-3902) | 127(14-271) | 5.3(1.5-9.4) |
|  | Saint Lucia | 0(0-0) | 57.8(49.8-65.7) | 0(0-0) | 36(0-214) | 2(0-14) | 5.1(1.3-7.5) |
| Eastern Europe and Central Asia | Republic of Moldova | 4(0-8) | 57.8(49.8-65.7) | 2(0-5) | 735(556-916) | 47(31-68) | 6.4(4.7-8.5) |
|  | Belarus | 27(15-39) | 57.8(49.8-65.7) | 15(8-23) | 1721(1460-1981) | 88(64-121) | 5.2(3.8-7) |
|  | Azerbaijan | 52(0-442) | 57.8(49.8-65.7) | 31(0-257) | 1073(370-1961) | 43(1-270) | 4.2(0.3-24.9) |
|  | Georgia | 3(0-25) | 57.8(49.8-65.7) | 2(0-14) | 636(192-1368) | 22(1-59) | 3.4(0.6-5.6) |
|  | Tajikistan | 3(0-25) | 57.8(49.8-65.7) | 2(0-15) | 479(123-1970) | 14(1-71) | 2.9(0.5-6.3) |
|  | Kyrgyzstan | 10(0-85) | 57.8(49.8-65.7) | 6(0-49) | 599(273-1099) | 17(2-60) | 2.5(0.7-10.1) |
|  | Armenia | 0(0-0) | 57.8(49.8-65.7) | 0(0-0) | 267(28-1212) | 7(0-51) | 2.4(0.4-4.7) |
|  | Kazakhstan | 26(0-101) | 57.8(49.8-65.7) | 15(0-58) | 2331(2004-2655) | 53(28-99) | 2.3(1.3-4.1) |
|  | Uzbekistan | 52(0-275) | 57.8(49.8-65.7) | 30(0-160) | 3326(2234-4468) | 79(24-211) | 2.3(1-6) |
|  | Albania | 4(0-11) | 57.8(49.8-65.7) | 2(0-7) | 274(207-343) | 4(1-9) | 1.3(0.3-2.7) |
|  | Montenegro | 0(0-0) | 57.8(49.8-65.7) | 0(0-0) | 115(63-168) | 1(0-2) | 0.8(0.5-1.2) |
|  | North Macedonia | 0(0-0) | 57.8(49.8-65.7) | 0(0-0) | 189(117-260) | 1(0-1) | 0.3(0.2-0.4) |
|  | Bosnia and Herzegovina | 0(0-0) | 57.8(49.8-65.7) | 0(0-0) | 476(362-589) | 1(1-2) | 0.2(0.1-0.3) |
| East and Southern Africa | Namibia | 324(167-484) | 86.6(79.8-92) | 280(143-422) | 826(582-1073) | 407(255-571) | 49.5(37.5-60) |
|  | Botswana | 231(106-358) | 86.6(79.8-92) | 199(91-310) | 782(474-1097) | 378(203-574) | 48.3(39.9-56.6) |
|  | Eswatini | 96(3-187) | 86.6(79.8-92) | 83(2-163) | 542(393-732) | 257(155-376) | 47.4(35.8-57.1) |
|  | Mozambique | 3990(3338-4615) | 86.6(79.8-92) | 3446(2842-4044) | 10812(9896-11724) | 5102(4236-5986) | 47.2(40.5-53.8) |
|  | Zambia | 2432(1948-2917) | 86.6(79.8-92) | 2096(1663-2549) | 6484(5833-7117) | 2998(2464-3564) | 46.3(40-52.5) |
|  | Lesotho | 147(0-1200) | 86.6(79.8-92) | 127(0-1036) | 846(508-1897) | 356(147-1263) | 43.5(25.6-68.9) |
|  | South Africa | 3958(0-18607) | 86.6(79.8-92) | 3407(0-16068) | 17749(13137-32596) | 7565(3162-20356) | 42.9(22.9-63.9) |
|  | Uganda | 4046(3461-4640) | 86.6(79.8-92) | 3494(2950-4066) | 13251(12314-14189) | 4725(3967-5570) | 35.6(30.7-41.3) |
|  | Zimbabwe | 1102(808-1387) | 86.6(79.8-92) | 949(691-1211) | 5689(5088-6287) | 2021(1538-2556) | 35.5(27.8-43.7) |
|  | Malawi | 1992(1528-2447) | 86.6(79.8-92) | 1721(1307-2135) | 8004(7189-8807) | 2718(2135-3355) | 34(27.8-40.6) |
|  | United Republic of Tanzania | 1389(922-1875) | 86.6(79.8-92) | 1199(793-1628) | 13608(12538-14682) | 2473(1849-3198) | 18.2(13.9-23) |
|  | Angola | 661(0-3008) | 86.6(79.8-92) | 569(0-2607) | 4275(3508-6729) | 708(78-2842) | 16.5(2.2-42.3) |
|  | Kenya | 660(451-871) | 86.6(79.8-92) | 570(387-756) | 8188(7502-8885) | 1274(929-1726) | 15.6(11.4-20.8) |
|  | Rwanda | 133(30-234) | 86.6(79.8-92) | 114(26-204) | 1315(1045-1590) | 197(100-303) | 15(8.2-21.7) |
|  | South Sudan | 174(0-1330) | 86.6(79.8-92) | 151(0-1146) | 1449(549-3776) | 216(17-1230) | 14.2(2.5-56.9) |
|  | Ethiopia | 163(0-371) | 86.6(79.8-92) | 141(0-322) | 11910(11023-12781) | 433(218-715) | 3.6(1.9-5.9) |
|  | Eritrea | 2(1-3) | 86.6(79.8-92) | 2(1-3) | 329(241-416) | 7(4-12) | 2.1(1.3-3.6) |
|  | Mauritius | 0(0-0) | 86.6(79.8-92) | 0(0-0) | 183(94-287) | 4(1-10) | 2.1(1.1-4.1) |
|  | Madagascar | 7(7-7) | 86.6(79.8-92) | 6(6-6) | 4977(4209-5731) | 45(26-77) | 0.9(0.5-1.5) |
|  | Comoros | 0(0-0) | 86.6(79.8-92) | 0(0-0) | 194(162-226) | 0(0-0) | 0.1(0-0.1) |
| Latin America | Panama | 39(0-319) | 62.5(48.4-75.4) | 24(0-200) | 687(152-1453) | 81(3-263) | 10.7(1.6-35.1) |
|  | Colombia | 433(0-3713) | 62.5(48.4-75.4) | 263(0-2352) | 9247(6342-12964) | 735(279-2826) | 8.1(3.9-23.9) |
|  | Uruguay | 12(0-59) | 62.5(48.4-75.4) | 7(0-38) | 878(648-1117) | 67(38-108) | 7.7(4.9-11.3) |
|  | Chile | 36(0-324) | 62.5(48.4-75.4) | 22(0-206) | 3190(1470-4927) | 204(58-418) | 6.2(3.3-11.3) |
|  | Costa Rica | 18(0-89) | 62.5(48.4-75.4) | 11(0-56) | 779(589-965) | 48(24-96) | 6.2(3.6-11.2) |
|  | Peru | 322(0-2686) | 62.5(48.4-75.4) | 197(0-1726) | 9317(6002-12994) | 562(195-2092) | 6(2.9-18.7) |
|  | Paraguay | 63(0-504) | 62.5(48.4-75.4) | 39(0-318) | 1648(808-2990) | 101(8-376) | 5.5(1-20.2) |
|  | Argentina | 236(0-2039) | 62.5(48.4-75.4) | 144(0-1294) | 8752(5355-13710) | 466(102-1961) | 5.3(1.9-14.3) |
|  | Venezuela | 195(0-1679) | 62.5(48.4-75.4) | 119(0-1048) | 6452(3746-9200) | 346(74-1269) | 5.2(1.8-17) |
|  | Mexico | 510(0-4074) | 62.5(48.4-75.4) | 312(0-2606) | 20353(13921-27107) | 1067(407-3315) | 5.1(2.6-14.5) |
|  | Ecuador | 74(0-575) | 62.5(48.4-75.4) | 45(0-356) | 3398(1944-4908) | 173(55-490) | 4.9(2.5-13.2) |
|  | El Salvador | 3(0-24) | 62.5(48.4-75.4) | 2(0-16) | 817(373-1519) | 26(4-71) | 3.2(1-5.3) |
|  | Nicaragua | 17(0-134) | 62.5(48.4-75.4) | 10(0-84) | 1044(400-1987) | 32(2-108) | 2.8(0.5-11.2) |
|  | Honduras | 4(0-15) | 62.5(48.4-75.4) | 2(0-10) | 1436(1103-1766) | 36(22-54) | 2.5(1.8-3.4) |
|  | Guatemala | 0(0-0) | 62.5(48.4-75.4) | 0(0-0) | 2124(1267-3137) | 31(9-67) | 1.5(0.6-2.3) |
|  | Bolivia | 0(0-0) | 62.5(48.4-75.4) | 0(0-0) | 2494(1619-4056) | 35(10-109) | 1.4(0.6-2.9) |
| Middle East and North Africa | Djibouti | 0(0-0) | 10.8(0.6-41.6) | 0(0-0) | 116(75-156) | 9(4-17) | 8(5.3-11.6) |
|  | Egypt | 80(0-535) | 10.8(0.6-41.6) | 5(0-112) | 10293(9120-11488) | 782(562-1087) | 7.6(5.6-10.3) |
|  | Sudan | 74(0-348) | 10.8(0.6-41.6) | 5(0-76) | 2860(2268-3469) | 64(36-137) | 2.2(1.3-4.5) |
|  | Libya | 16(0-118) | 10.8(0.6-41.6) | 1(0-24) | 642(354-930) | 12(5-34) | 1.8(1.2-5.1) |
|  | Iran (Islamic Republic of) | 170(0-420) | 10.8(0.6-41.6) | 14(0-106) | 5098(4432-5755) | 80(40-170) | 1.6(0.8-3.3) |
|  | Oman | 12(0-40) | 10.8(0.6-41.6) | 1(0-9) | 370(200-542) | 6(3-14) | 1.5(1-3.7) |
|  | Tunisia | 67(0-457) | 10.8(0.6-41.6) | 4(0-95) | 1382(926-1898) | 20(9-110) | 1.4(0.8-6.9) |
|  | Lebanon | 11(0-58) | 10.8(0.6-41.6) | 1(0-12) | 805(551-1054) | 11(6-23) | 1.3(0.9-2.7) |
|  | Yemen | 13(0-28) | 10.8(0.6-41.6) | 1(0-8) | 1080(812-1341) | 13(8-22) | 1.2(0.8-1.9) |
|  | Morocco | 108(0-339) | 10.8(0.6-41.6) | 8(0-80) | 5136(4507-5786) | 56(35-128) | 1.1(0.7-2.4) |
|  | Algeria | 61(0-264) | 10.8(0.6-41.6) | 4(0-58) | 4099(3531-4673) | 43(26-104) | 1(0.7-2.5) |
|  | Qatar | 7(0-39) | 10.8(0.6-41.6) | 0(0-8) | 132(5-286) | 1(0-9) | 0.9(0.3-12.5) |
|  | Saudi Arabia | 79(0-267) | 10.8(0.6-41.6) | 6(0-63) | 2329(1882-2800) | 21(11-78) | 0.9(0.5-3.2) |
|  | Somalia | 79(0-616) | 10.8(0.6-41.6) | 4(0-125) | 1734(1510-2271) | 17(9-136) | 0.9(0.5-6.6) |
|  | Kuwait | 5(0-25) | 10.8(0.6-41.6) | 0(0-5) | 326(172-476) | 2(1-7) | 0.6(0.4-2.2) |
|  | Iraq | 51(0-119) | 10.8(0.6-41.6) | 4(0-29) | 2297(1883-2706) | 8(3-33) | 0.4(0.1-1.4) |
|  | Jordan | 14(0-72) | 10.8(0.6-41.6) | 1(0-15) | 696(470-928) | 2(1-16) | 0.3(0.1-2.3) |
|  | Syrian Arab Republic | 22(0-191) | 10.8(0.6-41.6) | 1(0-40) | 1176(454-1897) | 2(0-41) | 0.2(0.1-3.7) |
| West and Central Africa | Gabon | 55(0-130) | 72.3(58.9-83.6) | 40(0-95) | 404(200-628) | 98(18-198) | 24(8.7-33.9) |
|  | Togo | 95(0-809) | 72.3(58.9-83.6) | 68(0-585) | 959(423-1966) | 208(16-719) | 20.6(3.6-45.8) |
|  | Burundi | 430(0-3263) | 72.3(58.9-83.6) | 308(0-2381) | 2261(1182-5742) | 418(28-2642) | 18.4(2.4-46.7) |
|  | Côte d'Ivoire | 214(93-334) | 72.3(58.9-83.6) | 153(66-248) | 3533(2953-4104) | 615(379-907) | 17.3(12.1-23.8) |
|  | Congo | 15(0-85) | 72.3(58.9-83.6) | 11(0-62) | 463(320-680) | 68(20-163) | 14.8(5.8-25.8) |
|  | Nigeria | 1536(1068-1992) | 72.3(58.9-83.6) | 1099(733-1495) | 20408(18672-22101) | 2875(2045-3932) | 14.1(10.5-18.7) |
|  | Guinea-Bissau | 6(0-49) | 72.3(58.9-83.6) | 4(0-35) | 273(21-583) | 34(1-108) | 12.8(4.7-21.3) |
|  | Ghana | 133(0-300) | 72.3(58.9-83.6) | 95(0-220) | 4580(3891-5311) | 559(319-871) | 12.2(7.8-17.5) |
|  | Cabo Verde | 5(0-23) | 72.3(58.9-83.6) | 4(0-17) | 63(32-112) | 7(0-23) | 10.5(1.4-21.3) |
|  | Burkina Faso | 93(0-420) | 72.3(58.9-83.6) | 66(0-307) | 1461(1342-1814) | 132(47-378) | 9.1(3.5-21) |
|  | Chad | 33(1-65) | 72.3(58.9-83.6) | 24(1-47) | 1494(1291-1697) | 121(73-185) | 8.1(5.2-11.9) |
|  | Gambia | 6(0-35) | 72.3(58.9-83.6) | 4(0-25) | 370(246-562) | 28(6-78) | 7.7(2.3-14.8) |
|  | Liberia | 27(0-238) | 72.3(58.9-83.6) | 19(0-170) | 892(605-1385) | 65(14-249) | 7.3(2.1-18.3) |
|  | Mali | 113(0-247) | 72.3(58.9-83.6) | 81(0-182) | 2958(2428-3516) | 209(79-371) | 7(3.2-11) |
|  | Guinea | 37(0-241) | 72.3(58.9-83.6) | 26(0-175) | 2863(2358-3532) | 193(74-423) | 6.8(2.9-12.4) |
|  | Benin | 24(0-141) | 72.3(58.9-83.6) | 17(0-103) | 898(620-1274) | 57(15-167) | 6.4(2.2-13.4) |
|  | Sierra Leone | 0(0-0) | 72.3(58.9-83.6) | 0(0-0) | 554(325-782) | 31(14-61) | 5.8(3.2-9.9) |
|  | Democratic Republic of the Congo | 168(0-1312) | 72.3(58.9-83.6) | 120(0-935) | 11029(4495-30751) | 562(54-3407) | 5.1(0.9-12) |
|  | Senegal | 82(0-630) | 72.3(58.9-83.6) | 59(0-456) | 2505(7-5580) | 108(0-565) | 4.4(0.8-10.3) |
|  | Niger | 28(0-197) | 72.3(58.9-83.6) | 20(0-142) | 1016(693-1481) | 38(9-169) | 3.8(1.1-11.6) |
|  | Mauritania | 19(0-177) | 72.3(58.9-83.6) | 14(0-129) | 580(90-1294) | 21(0-145) | 3.6(0.3-11.4) |
| Western and Central Europe and North America | Estonia | 0(0-0) | 43.1(31-55.6) | 0(0-0) | 377(299-455) | 40(27-58) | 10.7(7.6-14.7) |
|  | Portugal | 102(20-186) | 43.1(31-55.6) | 43(9-86) | 3433(2951-3916) | 351(246-486) | 10.3(7.3-13.9) |
|  | United States | 1109(1015-1202) | 43.1(31-55.6) | 478(338-625) | 93000(91634-94406) | 8732(6173-12198) | 9.4(6.6-13.1) |
|  | Spain | 293(157-427) | 43.1(31-55.6) | 125(62-203) | 11385(10287-12466) | 935(657-1300) | 8.2(5.9-11.3) |
|  | France | 223(96-352) | 43.1(31-55.6) | 95(39-163) | 20387(19028-21778) | 1630(1141-2302) | 8(5.6-11.2) |
|  | Italy | 876(680-1074) | 43.1(31-55.6) | 375(252-523) | 18942(17541-20360) | 1449(1073-1943) | 7.6(5.7-10.3) |
|  | Latvia | 0(0-0) | 43.1(31-55.6) | 0(0-0) | 491(340-644) | 29(16-47) | 5.9(4.1-8.3) |
|  | Switzerland | 29(13-45) | 43.1(31-55.6) | 12(5-21) | 2198(1768-2637) | 124(82-183) | 5.7(4-7.9) |
|  | Luxembourg | 0(0-0) | 43.1(31-55.6) | 0(0-0) | 115(44-188) | 6(3-11) | 5.7(3.9-8.3) |
|  | Malta | 5(0-16) | 43.1(31-55.6) | 2(0-7) | 149(112-188) | 9(5-15) | 5.7(3.3-9.2) |
|  | Israel | 91(16-166) | 43.1(31-55.6) | 39(7-76) | 2097(1716-2489) | 101(61-149) | 4.8(3.1-6.9) |
|  | Ireland | 6(1-11) | 43.1(31-55.6) | 3(0-5) | 1277(1116-1437) | 54(37-79) | 4.2(2.9-6.1) |
|  | Greece | 44(18-71) | 43.1(31-55.6) | 19(7-33) | 2181(1870-2481) | 88(62-124) | 4(2.9-5.6) |
|  | Netherlands | 37(26-48) | 43.1(31-55.6) | 16(10-23) | 5177(4838-5515) | 201(141-284) | 3.9(2.7-5.5) |
|  | Lithuania | 8(1-15) | 43.1(31-55.6) | 3(0-7) | 611(477-740) | 16(11-24) | 2.7(1.8-3.9) |
|  | Iceland | 0(0-0) | 43.1(31-55.6) | 0(0-0) | 85(57-113) | 2(1-3) | 2.4(1.6-3.5) |
|  | Romania | 58(29-87) | 43.1(31-55.6) | 25(12-41) | 5189(4558-5804) | 83(59-115) | 1.6(1.2-2.2) |
|  | Czechia | 16(6-26) | 43.1(31-55.6) | 7(2-12) | 2438(2218-2657) | 29(19-41) | 1.2(0.8-1.6) |
|  | Serbia | 16(0-38) | 43.1(31-55.6) | 7(0-17) | 1879(1529-2227) | 23(13-36) | 1.2(0.7-1.9) |
|  | Slovenia | 1(0-3) | 43.1(31-55.6) | 0(0-2) | 598(506-692) | 7(4-10) | 1.2(0.8-1.7) |
|  | Bulgaria | 11(4-18) | 43.1(31-55.6) | 5(2-8) | 1379(1195-1563) | 13(7-22) | 1(0.6-1.4) |
|  | Slovakia | 5(1-9) | 43.1(31-55.6) | 2(0-4) | 1245(1008-1481) | 8(5-12) | 0.7(0.4-1) |

Table S7. Estimated age-standardised incidence rate attributable to HIV of AIDS-defining cancers in 2022, by country

| UNAIDS region | Country | Cervical cancer | | | Non-Hodgkin lymphoma | | | Kaposi sarcoma | | | AIDS-defining cancers | | | Cancer burden* |
| --- | --- | --- | --- | --- | --- | --- | --- | --- | --- | --- | --- | --- | --- | --- |
|  |  | ASIR | PAF | ASIR attributable to HIV | ASIR | PAF | ASIR attributable to HIV | ASIR | PAF | ASIR attributable to HIV | ASIR | PAF | ASIR attributable to HIV |  |
| Asia and the Pacific | Papua New Guinea | 27.8 | 10.1 | 2.80 | 6.6 | 19.8 | 1.31 | 0.1 | 26.3 | 0.02 | 24.7 | 13.5 | 3.33 | Group 1 |
|  | Thailand | 14.9 | 6.6 | 0.99 | 5.9 | 19.2 | 1.13 | 0.1 | 26.3 | 0.02 | 16.4 | 12.1 | 1.99 | Group 1 |
|  | Fiji | 35.0 | 3.1 | 1.10 | 5.3 | 9.0 | 0.48 | 0 | 26.3 | 0 | 27.6 | 4.6 | 1.28 | Group 2 |
|  | Cambodia | 15.2 | 5.1 | 0.78 | 4.3 | 13.7 | 0.59 | 0 | 26.3 | 0 | 15.0 | 8.0 | 1.20 | Group 4 |
|  | Myanmar | 21.4 | 4.6 | 0.98 | 3.4 | 14.2 | 0.48 | 0 | 26.3 | 0 | 17.7 | 6.7 | 1.19 | Group 2 |
|  | Indonesia | 23.3 | 1.5 | 0.36 | 5.5 | 6.2 | 0.34 | 0 | 26.3 | 0 | 20.7 | 3.0 | 0.62 | Group 2 |
|  | Malaysia | 10.3 | 1.1 | 0.11 | 6.0 | 7.4 | 0.45 | 0.1 | 26.3 | 0.02 | 13.3 | 4.4 | 0.59 | Group 4 |
|  | Lao People's Democratic Republic | 12.0 | 2.2 | 0.27 | 4.3 | 7.9 | 0.34 | 0 | 26.3 | 0 | 12.4 | 4.5 | 0.56 | Group 4 |
|  | Australia | 5.3 | 0.3 | 0.02 | 12.4 | 3.3 | 0.41 | 0.1 | 26.3 | 0.03 | 18.1 | 3.1 | 0.56 | Group 2 |
|  | Singapore | 7.4 | 0.2 | 0.02 | 11.3 | 3.5 | 0.39 | 0.1 | 26.3 | 0.02 | 18.0 | 2.8 | 0.51 | Group 2 |
|  | Viet Nam | 7.1 | 1.7 | 0.12 | 3.0 | 7.4 | 0.22 | 0.1 | 26.3 | 0.04 | 8.2 | 4.6 | 0.38 | Group 4 |
|  | New Zealand | 4.9 | 0.2 | 0.01 | 10.3 | 2.1 | 0.22 | 0.1 | 26.3 | 0.02 | 15.4 | 2.0 | 0.31 | Group 4 |
|  | Timor-Leste | 14.8 | 1.2 | 0.18 | 3.7 | 4.3 | 0.16 | 0 | 26.3 | 0 | 13.2 | 2.2 | 0.30 | Group 4 |
|  | Philippines | 15.5 | 0.2 | 0.04 | 4.9 | 4.7 | 0.23 | 0 | 26.3 | 0 | 15.4 | 1.9 | 0.30 | Group 4 |
|  | Pakistan | 5.4 | 0.6 | 0.03 | 4.1 | 4.0 | 0.16 | 0 | 26.3 | 0 | 8.2 | 2.9 | 0.24 | Group 4 |
|  | Bhutan | 13.6 | 1.6 | 0.22 | 1.4 | 4.6 | 0.07 | 0 | 26.3 | 0 | 9.3 | 2.1 | 0.19 | Group 4 |
|  | Nepal | 14.2 | 1.0 | 0.14 | 2.1 | 3.2 | 0.07 | 0 | 26.3 | 0 | 11.8 | 1.4 | 0.17 | Group 4 |
|  | Afghanistan | 10.3 | 0.1 | 0.01 | 2.2 | 0.3 | 0.01 | 0.1 | 26.3 | 0.03 | 8.9 | 0.5 | 0.04 | Group 4 |
|  | Sri Lanka | 9.2 | 0.1 | 0.01 | 3.3 | 0.7 | 0.02 | 0 | 26.3 | 0 | 9.8 | 0.3 | 0.03 | Group 4 |
|  | Mongolia | 20.2 | 0.1 | 0.02 | 1.5 | 0.7 | 0.01 | 0 | 26.3 | 0 | 14.7 | 0.1 | 0.02 | Group 4 |
|  | Bangladesh | 11.3 | 0.1 | 0.01 | 2.0 | 0.3 | 0.01 | 0 | 26.3 | 0 | 9.1 | 0.1 | 0.01 | Group 4 |
| Caribbean | Suriname | 24.2 | 5.4 | 1.30 | 5.6 | 23.8 | 1.33 | 0.3 | 57.8 | 0.16 | 22.0 | 14.4 | 3.18 | Group 1 |
|  | Guyana | 30.3 | 6.3 | 1.91 | 5.9 | 26.2 | 1.55 | 0 | 57.8 | 0 | 25.6 | 17.2 | 3.00 | Group 1 |
|  | Barbados | 13.8 | 2.6 | 0.35 | 7.6 | 15.1 | 1.15 | 2.0 | 57.8 | 1.16 | 20.1 | 14.0 | 2.82 | Group 1 |
|  | Bahamas | 15.2 | 4.0 | 0.60 | 6.2 | 19.6 | 1.22 | 1.0 | 57.8 | 0.58 | 18.2 | 14.8 | 2.70 | Group 1 |
|  | Haiti | 16.6 | 6.9 | 1.14 | 4.4 | 25.1 | 1.11 | 0.5 | 57.8 | 0.31 | 16.4 | 16.3 | 2.67 | Group 1 |
|  | Jamaica | 20.4 | 3.9 | 0.79 | 6.9 | 18.1 | 1.25 | 0 | 57.8 | 0 | 20.8 | 9.1 | 1.90 | Group 1 |
|  | Dominican Republic | 15.6 | 3.5 | 0.55 | 2.6 | 16.4 | 0.43 | 0.3 | 57.8 | 0.17 | 13.0 | 8.7 | 1.13 | Group 3 |
|  | Belize | 17.3 | 4.1 | 0.70 | 2.2 | 19.4 | 0.43 | 0 | 57.8 | 0 | 13.2 | 7.5 | 0.99 | Group 4 |
|  | Saint Lucia | 15.7 | 1.6 | 0.26 | 7.3 | 8.5 | 0.62 | 0 | 57.8 | 0 | 18.3 | 5.1 | 0.93 | Group 2 |
|  | Cuba | 12.8 | 0.6 | 0.08 | 6.4 | 8.2 | 0.53 | 0.1 | 57.8 | 0.06 | 15.5 | 5.3 | 0.82 | Group 4 |
| Eastern Europe and Central Asia | Republic of Moldova | 14.2 | 1.8 | 0.26 | 5.8 | 12.0 | 0.69 | 0.1 | 57.8 | 0.04 | 15.8 | 6.4 | 1.01 | Group 2 |
|  | Belarus | 9.3 | 0.9 | 0.08 | 6.4 | 6.6 | 0.42 | 0.2 | 57.8 | 0.09 | 13.5 | 5.2 | 0.70 | Group 4 |
|  | Georgia | 10.4 | 0.6 | 0.06 | 5.0 | 5.6 | 0.28 | 0.1 | 57.8 | 0.04 | 12.6 | 3.4 | 0.43 | Group 4 |
|  | Azerbaijan | 8.2 | 0.3 | 0.02 | 3.6 | 2.5 | 0.09 | 0.4 | 57.8 | 0.25 | 10.0 | 4.2 | 0.42 | Group 4 |
|  | Kazakhstan | 15.5 | 0.7 | 0.11 | 2.2 | 5.3 | 0.12 | 0.1 | 57.8 | 0.06 | 12.6 | 2.3 | 0.29 | Group 4 |
|  | Kyrgyzstan | 14.1 | 0.7 | 0.10 | 1.6 | 4.6 | 0.07 | 0.2 | 57.8 | 0.09 | 11.1 | 2.5 | 0.28 | Group 4 |
|  | Uzbekistan | 14.8 | 0.8 | 0.11 | 1.8 | 4.3 | 0.08 | 0.2 | 57.8 | 0.09 | 11.5 | 2.3 | 0.27 | Group 4 |
|  | Tajikistan | 6.6 | 0.5 | 0.04 | 2.3 | 4.1 | 0.09 | 0 | 57.8 | 0 | 6.8 | 2.9 | 0.20 | Group 4 |
|  | Armenia | 6.8 | 0.6 | 0.04 | 3.0 | 5.1 | 0.15 | 0 | 57.8 | 0 | 8.0 | 2.4 | 0.20 | Group 4 |
|  | Montenegro | 12.0 | 0.1 | 0.01 | 5.6 | 1.6 | 0.09 | 0 | 57.8 | 0 | 14.0 | 0.8 | 0.12 | Group 4 |
|  | Albania | 8.7 | 0.1 | 0.01 | 2.3 | 1.2 | 0.03 | 0.1 | 57.8 | 0.04 | 8.1 | 1.3 | 0.11 | Group 4 |
|  | Bosnia and Herzegovina | 12.3 | 0.0 | 0.00 | 3.6 | 0.4 | 0.02 | 0 | 57.8 | 0 | 11.7 | 0.2 | 0.02 | Group 4 |
|  | North Macedonia | 6.7 | 0.0 | 0.00 | 2.3 | 0.7 | 0.02 | 0 | 57.8 | 0 | 6.8 | 0.3 | 0.02 | Group 4 |
| East and Southern Africa | Eswatini | 95.9 | 38.2 | 36.62 | 3.1 | 50.3 | 1.56 | 8.8 | 86.6 | 7.62 | 73.6 | 47.4 | 34.89 | Group 1 |
|  | Zambia | 71.5 | 20.9 | 14.97 | 3.5 | 33.2 | 1.16 | 15.7 | 86.6 | 13.59 | 69.1 | 46.3 | 32.00 | Group 1 |
|  | Mozambique | 47.8 | 21.7 | 10.36 | 5.6 | 34.4 | 1.93 | 14.4 | 86.6 | 12.47 | 54.8 | 47.2 | 25.89 | Group 1 |
|  | Zimbabwe | 68.2 | 21.1 | 14.42 | 10.3 | 30.3 | 3.12 | 9.5 | 86.6 | 8.23 | 69.0 | 35.5 | 24.53 | Group 1 |
|  | Namibia | 33.5 | 21.7 | 7.26 | 7.4 | 34.0 | 2.52 | 13.6 | 86.6 | 11.78 | 46.9 | 49.5 | 23.24 | Group 1 |
|  | Malawi | 70.9 | 14.8 | 10.52 | 6.5 | 22.3 | 1.45 | 12.0 | 86.6 | 10.39 | 66.8 | 34.0 | 22.69 | Group 1 |
|  | Lesotho | 60.5 | 32.2 | 19.46 | 2.6 | 43.3 | 1.13 | 6.5 | 86.6 | 5.63 | 49.4 | 43.5 | 21.49 | Group 1 |
|  | Botswana | 39.1 | 31.0 | 12.12 | 4.3 | 41.6 | 1.79 | 9.3 | 86.6 | 8.05 | 41.6 | 48.3 | 20.10 | Group 1 |
|  | Uganda | 53.8 | 11.9 | 6.40 | 7.4 | 17.3 | 1.28 | 11.1 | 86.6 | 9.61 | 56.2 | 35.6 | 20.02 | Group 1 |
|  | South Africa | 33.2 | 28.2 | 9.37 | 5.6 | 37.2 | 2.08 | 6.0 | 86.6 | 5.20 | 34.7 | 42.9 | 14.88 | Group 1 |
|  | United Republic of Tanzania | 64.8 | 9.9 | 6.42 | 3.3 | 14.1 | 0.47 | 3.4 | 86.6 | 2.94 | 49.3 | 18.2 | 8.98 | Group 1 |
|  | Kenya | 32.8 | 8.5 | 2.80 | 4.8 | 12.1 | 0.58 | 1.5 | 86.6 | 1.30 | 28.2 | 15.6 | 4.40 | Group 1 |
|  | Angola | 30.4 | 3.5 | 1.06 | 2.8 | 5.0 | 0.14 | 2.8 | 86.6 | 2.42 | 25.7 | 16.5 | 4.25 | Group 1 |
|  | South Sudan | 21.4 | 3.5 | 0.75 | 3.5 | 5.3 | 0.19 | 2.0 | 86.6 | 1.73 | 19.9 | 14.2 | 2.84 | Group 1 |
|  | Rwanda | 18.9 | 6.1 | 1.15 | 3.0 | 9.1 | 0.27 | 1.2 | 86.6 | 1.04 | 16.9 | 15.0 | 2.54 | Group 1 |
|  | Ethiopia | 22.3 | 2.1 | 0.48 | 4.1 | 3.1 | 0.13 | 0.2 | 86.6 | 0.16 | 19.0 | 3.6 | 0.69 | Group 2 |
|  | Eritrea | 16.4 | 1.3 | 0.22 | 4.9 | 2.1 | 0.10 | 0.1 | 86.6 | 0.06 | 16.2 | 2.1 | 0.35 | Group 2 |
|  | Madagascar | 41.8 | 0.7 | 0.28 | 4.1 | 1.3 | 0.05 | 0 | 86.6 | 0 | 30.7 | 0.9 | 0.28 | Group 2 |
|  | Mauritius | 12.9 | 1.6 | 0.20 | 2.5 | 3.9 | 0.10 | 0 | 86.6 | 0 | 11.1 | 2.1 | 0.23 | Group 4 |
|  | Comoros | 52.0 | 0.1 | 0.03 | 4.2 | 0.1 | 0.01 | 0 | 86.6 | 0 | 37.1 | 0.1 | 0.03 | Group 2 |
| Latin America | Peru | 23.9 | 0.7 | 0.17 | 9.6 | 7.9 | 0.76 | 0.8 | 62.5 | 0.49 | 26.8 | 6.0 | 1.61 | Group 2 |
|  | Panama | 14.5 | 1.7 | 0.25 | 4.5 | 15.2 | 0.68 | 0.7 | 62.5 | 0.46 | 15.0 | 10.7 | 1.60 | Group 3 |
|  | Uruguay | 15.9 | 1.5 | 0.23 | 8.7 | 10.7 | 0.93 | 0.3 | 62.5 | 0.18 | 20.5 | 7.7 | 1.57 | Group 2 |
|  | Paraguay | 30.6 | 1.1 | 0.33 | 5.8 | 8.1 | 0.47 | 0.7 | 62.5 | 0.45 | 26.1 | 5.5 | 1.44 | Group 2 |
|  | Colombia | 13.7 | 0.7 | 0.10 | 6.0 | 10.7 | 0.64 | 0.7 | 62.5 | 0.43 | 16.5 | 8.1 | 1.34 | Group 2 |
|  | Venezuela | 22.7 | 0.9 | 0.20 | 6.2 | 8.0 | 0.50 | 0.6 | 62.5 | 0.39 | 22.3 | 5.2 | 1.16 | Group 2 |
|  | Argentina | 16.8 | 1.0 | 0.17 | 6.6 | 7.1 | 0.47 | 0.5 | 62.5 | 0.28 | 18.7 | 5.3 | 0.99 | Group 2 |
|  | Ecuador | 17.7 | 0.9 | 0.16 | 7.4 | 6.7 | 0.50 | 0.3 | 62.5 | 0.21 | 20.1 | 4.9 | 0.99 | Group 2 |
|  | Chile | 11.3 | 0.6 | 0.07 | 5.3 | 9.8 | 0.52 | 0.2 | 62.5 | 0.09 | 13.5 | 6.2 | 0.84 | Group 4 |
|  | Costa Rica | 10.6 | 0.5 | 0.05 | 5.7 | 8.2 | 0.47 | 0.2 | 62.5 | 0.15 | 13.5 | 6.2 | 0.83 | Group 4 |
|  | Mexico | 13.2 | 0.5 | 0.07 | 6.2 | 7.0 | 0.44 | 0.3 | 62.5 | 0.21 | 16.1 | 5.1 | 0.82 | Group 2 |
|  | Nicaragua | 20.6 | 0.5 | 0.11 | 4.6 | 4.3 | 0.20 | 0.2 | 62.5 | 0.14 | 19.0 | 2.8 | 0.52 | Group 2 |
|  | Honduras | 19.5 | 0.8 | 0.16 | 6.1 | 4.9 | 0.30 | 0.1 | 62.5 | 0.03 | 19.3 | 2.5 | 0.48 | Group 2 |
|  | El Salvador | 15.2 | 1.2 | 0.18 | 2.5 | 8.5 | 0.21 | 0 | 62.5 | 0 | 13.1 | 3.2 | 0.42 | Group 4 |
|  | Bolivia | 38.7 | 0.7 | 0.28 | 2.5 | 6.7 | 0.17 | 0 | 62.5 | 0 | 26.4 | 1.4 | 0.37 | Group 2 |
|  | Guatemala | 21.5 | 0.8 | 0.16 | 2.5 | 5.1 | 0.13 | 0 | 62.5 | 0 | 16.7 | 1.5 | 0.24 | Group 2 |
| Middle East and North Africa | Djibouti | 16.5 | 3.4 | 0.56 | 5.0 | 15.3 | 0.76 | 0 | 10.8 | 0 | 15.8 | 8.0 | 1.27 | Group 2 |
|  | Egypt | 2.8 | 1.2 | 0.04 | 9.6 | 8.5 | 0.81 | 0.1 | 10.8 | 0.01 | 13.3 | 7.6 | 1.01 | Group 4 |
|  | Sudan | 8.6 | 0.6 | 0.05 | 4.9 | 3.1 | 0.15 | 0.3 | 10.8 | 0.03 | 11.5 | 2.2 | 0.26 | Group 4 |
|  | Somalia | 26.6 | 0.4 | 0.09 | 4.3 | 1.6 | 0.07 | 0.7 | 10.8 | 0.08 | 22.3 | 0.9 | 0.21 | Group 2 |
|  | Libya | 8.0 | 0.4 | 0.03 | 5.3 | 2.5 | 0.14 | 0.3 | 10.8 | 0.03 | 11.5 | 1.8 | 0.21 | Group 4 |
|  | Oman | 6.4 | 0.2 | 0.02 | 6.3 | 1.6 | 0.10 | 0.2 | 10.8 | 0.03 | 11.2 | 1.5 | 0.17 | Group 4 |
|  | Lebanon | 3.6 | 0.1 | 0.00 | 8.4 | 1.4 | 0.12 | 0.1 | 10.8 | 0.01 | 12.4 | 1.3 | 0.16 | Group 4 |
|  | Morocco | 12.0 | 0.3 | 0.03 | 5.7 | 1.6 | 0.09 | 0.3 | 10.8 | 0.03 | 14.5 | 1.1 | 0.16 | Group 4 |
|  | Tunisia | 5.3 | 0.2 | 0.01 | 5.8 | 1.6 | 0.09 | 0.4 | 10.8 | 0.04 | 10.6 | 1.4 | 0.15 | Group 4 |
|  | Algeria | 8.0 | 0.3 | 0.02 | 4.9 | 1.5 | 0.07 | 0.1 | 10.8 | 0.01 | 10.8 | 1.0 | 0.11 | Group 4 |
|  | Iran (Islamic Republic of) | 2.5 | 0.2 | 0.01 | 4.0 | 1.6 | 0.07 | 0.2 | 10.8 | 0.02 | 6.5 | 1.6 | 0.10 | Group 4 |
|  | Saudi Arabia | 2.4 | 0.1 | 0.00 | 6.0 | 0.7 | 0.04 | 0.3 | 10.8 | 0.03 | 8.9 | 0.9 | 0.08 | Group 4 |
|  | Yemen | 2.1 | 0.3 | 0.01 | 3.6 | 1.3 | 0.05 | 0.1 | 10.8 | 0.01 | 5.7 | 1.2 | 0.07 | Group 4 |
|  | Qatar | 4.7 | 0.1 | 0.00 | 3.9 | 0.7 | 0.03 | 0.2 | 10.8 | 0.02 | 7.0 | 0.9 | 0.06 | Group 4 |
|  | Kuwait | 3.1 | 0.0 | 0.00 | 6.2 | 0.6 | 0.04 | 0.1 | 10.8 | 0.01 | 9.4 | 0.6 | 0.06 | Group 4 |
|  | Iraq | 2.2 | 0.0 | 0.00 | 6.6 | 0.2 | 0.01 | 0.2 | 10.8 | 0.02 | 9.4 | 0.4 | 0.04 | Group 4 |
|  | Jordan | 3.2 | 0.0 | 0.00 | 6.5 | 0.2 | 0.01 | 0.2 | 10.8 | 0.02 | 9.9 | 0.3 | 0.03 | Group 4 |
|  | Syrian Arab Republic | 2.5 | 0.0 | 0.00 | 5.5 | 0.1 | 0.01 | 0.2 | 10.8 | 0.02 | 8.3 | 0.2 | 0.01 | Group 4 |
| West and Central Africa | Burundi | 43.6 | 2.9 | 1.26 | 4.5 | 17.8 | 0.80 | 4.9 | 72.3 | 3.54 | 38.8 | 18.4 | 7.16 | Group 1 |
|  | Gabon | 32.5 | 9.8 | 3.19 | 3.9 | 40.0 | 1.56 | 2.9 | 72.3 | 2.10 | 27.7 | 24.0 | 6.65 | Group 1 |
|  | Côte d'Ivoire | 32.0 | 6.6 | 2.10 | 4.8 | 31.7 | 1.52 | 1.1 | 72.3 | 0.80 | 25.8 | 17.3 | 4.47 | Group 1 |
|  | Togo | 19.1 | 5.1 | 0.98 | 3.8 | 27.4 | 1.04 | 1.4 | 72.3 | 1.01 | 18.0 | 20.6 | 3.71 | Group 1 |
|  | Guinea-Bissau | 34.3 | 6.9 | 2.37 | 2.8 | 34.2 | 0.96 | 0.3 | 72.3 | 0.23 | 26.1 | 12.8 | 3.33 | Group 1 |
|  | Nigeria | 26.2 | 4.1 | 1.08 | 4.0 | 23.1 | 0.93 | 1.0 | 72.3 | 0.72 | 22.2 | 14.1 | 3.13 | Group 1 |
|  | Ghana | 27.0 | 4.3 | 1.16 | 5.0 | 24.0 | 1.20 | 0.5 | 72.3 | 0.35 | 23.1 | 12.2 | 2.81 | Group 1 |
|  | Guinea | 55.0 | 3.8 | 2.07 | 2.6 | 22.2 | 0.58 | 0.4 | 72.3 | 0.31 | 40.8 | 6.8 | 2.77 | Group 2 |
|  | Congo | 22.3 | 9.0 | 2.00 | 1.2 | 39.7 | 0.48 | 0.4 | 72.3 | 0.25 | 15.8 | 14.8 | 2.33 | Group 1 |
|  | Mali | 43.1 | 2.5 | 1.10 | 2.6 | 15.4 | 0.40 | 0.9 | 72.3 | 0.62 | 31.3 | 7.0 | 2.20 | Group 2 |
|  | Liberia | 39.6 | 2.7 | 1.09 | 3.6 | 16.3 | 0.59 | 0.7 | 72.3 | 0.53 | 29.7 | 7.3 | 2.18 | Group 2 |
|  | Gambia | 39.4 | 4.2 | 1.66 | 1.7 | 24.6 | 0.42 | 0.3 | 72.3 | 0.21 | 26.7 | 7.7 | 2.05 | Group 2 |
|  | Chad | 23.5 | 3.0 | 0.71 | 2.5 | 18.2 | 0.46 | 0.3 | 72.3 | 0.24 | 17.8 | 8.1 | 1.44 | Group 1 |
|  | Cabo Verde | 16.1 | 2.1 | 0.33 | 2.3 | 17.1 | 0.39 | 0.9 | 72.3 | 0.64 | 13.7 | 10.5 | 1.44 | Group 3 |
|  | Burkina Faso | 15.9 | 1.9 | 0.30 | 2.3 | 12.1 | 0.28 | 0.6 | 72.3 | 0.46 | 13.8 | 9.1 | 1.25 | Group 3 |
|  | Democratic Republic of the Congo | 32.9 | 1.9 | 0.64 | 3.1 | 13.0 | 0.40 | 0.2 | 72.3 | 0.16 | 24.4 | 5.1 | 1.24 | Group 2 |
|  | Senegal | 34.3 | 1.0 | 0.34 | 2.4 | 7.5 | 0.18 | 0.6 | 72.3 | 0.43 | 26.1 | 4.4 | 1.16 | Group 2 |
|  | Benin | 18.1 | 2.2 | 0.40 | 2.1 | 13.7 | 0.29 | 0.3 | 72.3 | 0.19 | 14.2 | 6.4 | 0.91 | Group 4 |
|  | Mauritania | 28.3 | 0.5 | 0.14 | 2.6 | 4.5 | 0.12 | 0.6 | 72.3 | 0.40 | 21.5 | 3.6 | 0.78 | Group 2 |
|  | Sierra Leone | 16.5 | 3.6 | 0.60 | 0.6 | 21.3 | 0.14 | 0 | 72.3 | 0 | 10.8 | 5.8 | 0.62 | Group 4 |
|  | Niger | 9.3 | 0.5 | 0.05 | 1.8 | 4.0 | 0.07 | 0.1 | 72.3 | 0.09 | 8.0 | 3.8 | 0.30 | Group 4 |
| Western and Central Europe and North America | Portugal | 11.1 | 1.1 | 0.12 | 10.8 | 12.2 | 1.32 | 0.7 | 43.1 | 0.29 | 20.5 | 10.3 | 2.10 | Group 1 |
|  | Estonia | 11.8 | 1.8 | 0.21 | 9.4 | 14.9 | 1.40 | 0 | 43.1 | 0 | 18.3 | 10.7 | 1.96 | Group 1 |
|  | United States | 6.3 | 0.7 | 0.04 | 12.5 | 10.5 | 1.31 | 0.2 | 43.1 | 0.10 | 19.0 | 9.4 | 1.78 | Group 1 |
|  | France | 6.6 | 0.9 | 0.06 | 11.3 | 8.9 | 1.00 | 0.2 | 43.1 | 0.08 | 17.6 | 8.0 | 1.41 | Group 2 |
|  | Italy | 5.0 | 0.5 | 0.03 | 11.6 | 6.8 | 0.79 | 0.5 | 43.1 | 0.23 | 17.5 | 7.6 | 1.34 | Group 2 |
|  | Spain | 5.4 | 0.5 | 0.03 | 9.6 | 8.8 | 0.84 | 0.4 | 43.1 | 0.16 | 15.2 | 8.2 | 1.25 | Group 3 |
|  | Malta | 4.2 | 0.3 | 0.01 | 14.6 | 4.7 | 0.69 | 0.4 | 43.1 | 0.16 | 20.4 | 5.7 | 1.17 | Group 2 |
|  | Latvia | 16.9 | 1.0 | 0.17 | 7.7 | 10.5 | 0.81 | 0 | 43.1 | 0 | 19.5 | 5.9 | 1.15 | Group 2 |
|  | Israel | 6.2 | 0.3 | 0.02 | 13.9 | 3.6 | 0.50 | 0.6 | 43.1 | 0.25 | 21.0 | 4.8 | 1.02 | Group 2 |
|  | Switzerland | 4.1 | 0.5 | 0.02 | 9.8 | 5.8 | 0.57 | 0.2 | 43.1 | 0.08 | 14.4 | 5.7 | 0.82 | Group 4 |
|  | Ireland | 7.5 | 0.5 | 0.03 | 11.6 | 4.9 | 0.57 | 0.1 | 43.1 | 0.04 | 18.5 | 4.2 | 0.79 | Group 2 |
|  | Luxembourg | 4.4 | 0.6 | 0.03 | 8.9 | 6.8 | 0.60 | 0 | 43.1 | 0 | 13.2 | 5.7 | 0.75 | Group 4 |
|  | Netherlands | 6.7 | 0.2 | 0.02 | 12.0 | 4.2 | 0.50 | 0.1 | 43.1 | 0.06 | 18.5 | 3.9 | 0.72 | Group 2 |
|  | Greece | 5.5 | 0.2 | 0.01 | 7.4 | 4.1 | 0.30 | 0.1 | 43.1 | 0.04 | 12.3 | 4.0 | 0.50 | Group 4 |
|  | Iceland | 9.4 | 0.3 | 0.03 | 10.2 | 3.0 | 0.30 | 0 | 43.1 | 0 | 17.8 | 2.4 | 0.42 | Group 2 |
|  | Lithuania | 13.7 | 0.3 | 0.04 | 5.8 | 4.0 | 0.23 | 0.1 | 43.1 | 0.04 | 15.4 | 2.7 | 0.42 | Group 4 |
|  | Romania | 21.7 | 0.3 | 0.06 | 5.6 | 2.8 | 0.16 | 0.2 | 43.1 | 0.07 | 20.1 | 1.6 | 0.32 | Group 2 |
|  | Slovenia | 7.2 | 0.0 | 0.003 | 10.3 | 1.3 | 0.14 | 0 | 43.1 | 0 | 16.5 | 1.2 | 0.19 | Group 2 |
|  | Serbia | 13.4 | 0.1 | 0.01 | 6.1 | 1.6 | 0.10 | 0.1 | 43.1 | 0.06 | 15.6 | 1.2 | 0.19 | Group 4 |
|  | Czechia | 8.0 | 0.0 | 0.004 | 7.9 | 1.2 | 0.10 | 0.1 | 43.1 | 0.03 | 14.2 | 1.2 | 0.17 | Group 4 |
|  | Bulgaria | 15.4 | 0.1 | 0.01 | 4.0 | 1.6 | 0.07 | 0.1 | 43.1 | 0.02 | 14.1 | 1.0 | 0.14 | Group 4 |
|  | Slovakia | 12.9 | 0.0 | 0.004 | 6.3 | 0.9 | 0.05 | 0 | 43.1 | 0 | 15.4 | 0.7 | 0.10 | Group 4 |

* Group 1, 2, 3 and 4 represents countries having high cancer burden with high HIV attribution, high cancer burden with low HIV attribution, low cancer burden with high HIV attribution and low cancer burden with low HIV attribution, respectively.

Abbreviations: ASIR, age-standardised incidence rate; PAF, population attributable fraction

Appendix 2. Estimated AIDS-defining cancer burden in excluded countries having no HIV prevalence data

We performed additional analyses to estimate AIDS-defining cancer burden in excluded countries having no HIV prevalence data. These 31 excluded countries account for approximately 30.8% (2.46 billion people) of the global population (7.99 billion people) based on United Nations population estimates for the reference year. Of these people, 78.2% (approximately 1.93 billion people) are located in Asia and the Pacific, including China (1.43 billion), Japan (0.12 billion), the Republic of Korea (0.05 billion), and the Democratic People’s Republic of Korea (0.03 billion). Based on GLOBOCAN 2022 estimates, we calculated the proportion of global cancer burden represented by the 31 excluded countries for each cancer type. The excluded countries accounted for approximately 35.2% of global cervical cancer cases (233,115/662,044), 39.8% of global NHL cases (220,018/553,156), 11.9% of global KS cases (4,257/35,793), and 36.6% of global ADCs (457,390/1,250,993). Although the excluded countries include both high- and low- income settings, the overall excluded cancer burden for ADCs (36.6%) is proportionally greater than the excluded population share (30.8%), though this pattern varies by cancer type, being notably lower for KS (11.9%). The direction of resulting bias is not straightforward because HIV prevalence varies widely among the excluded nations, ranging from high in some African countries to low in many Asian and European countries. Nevertheless, our main analysis already includes the countries with the highest HIV prevalence (mostly in sub-Saharan Africa), so we believe the overall conclusions remain robust.

Records identified through database searching

(n=6102)

Records after duplicates removed (n=3884)

Records screened

(n=3884)

Records excluded (n = 3807) Title screening: 3440

Abstract screening: 367

Full-text articles excluded with reasons (n=64)

Wrong study design: 6

Wrong population: 5

Wrong outcome: 3

No relevant information: 22

Duplicate: 5

Only abstract: 17

Review: 1

Case report：2

book：1

cases≤4：2

Full-text articles assessed

for eligibility

(n=77)

Studies included in quantitative synthesis

(Meta-analysis)

(n=13)

Duplicates excluded (n = 2218)

Figure. S2. Flow chart of the search for cervical cancer and newly added studies

Records identified through database searching

(n=1440)

Records after duplicates removed (n=1267)

Records screened

(n=1267)

Records excluded (n = 1235)

Review: 91

Case: 111

Title: 935

Abstract: 97

Reports not retrieved: 1

Full-text articles excluded with reasons (n=27)

Review: 1

Meta-analysis: 2

Population overlapping: 1

No needed effect value: 9

Non-target population: 14

Full-text articles assessed

for eligibility

(n=32)

Studies included in quantitative synthesis

(Meta-analysis)

(n=5)

Duplicates excluded (n = 173)

Figure. S3. Flow chart of the search for non-Hodgkin lymphoma and newly added studies

Records identified through database searching

(n=364)

Records after duplicates removed (n=248)

Records screened

(n=248)

Records excluded (n = 211)

Review: 20

Duplicate population: 6

Only abstract: 1

Wrong study: 110

HIV patients only: 74

Studies included in quantitative synthesis

(Meta-analysis)

(n=37)

Duplicates excluded (n = 116)

Figure. S4. Flow chart of the search for Kaposi sarcoma and newly added studies

Figure. S5. Forest plot showing the pooled risk ratio of developing cervical cancer in women living with HIV

Figure. S6. Forest plot showing the pooled risk ratio of developing non-Hodgkin lymphoma in the population living with HIV

Figure. S7. Forest plot showing the pooled risk ratio of developing Kaposi sarcoma in the population living with HIV

Figure. S8. Population attributable fraction (PAF) of HIV for AIDS-defining cancers, stratified by UNAIDS regions

(A) PAF of HIV for cervical cancer in women living with HIV in 2022. (B) PAF of HIV for non-Hodgkin lymphoma in population living with HIV in 2022. (C) PAF of HIV for Kaposi sarcoma in population living with HIV in 2022.

Figure. S9. Estimated HIV-attributable and all-cause age-standardised incidence rate in the 50 highest-ranked countries
